# Computational Exploration of the Synergistic Anticancer Effect of a Multi-Action Ru(II)–Pt(IV) Conjugate

**DOI:** 10.1021/acs.inorgchem.2c02223

**Published:** 2022-07-28

**Authors:** Stefano Scoditti, Gloria Mazzone, Nico Sanna, Emilia Sicilia

**Affiliations:** † Dipartimento di Chimica e Tecnologie Chimiche, Università della Calabria, 87036 Rende, CS, Italy; ‡ Department for Innovation in Biology Agro-Food and Forest Systems (DIBAF), University of Tuscia, Largo dell’Università snc, 01100 Viterbo, Italy

## Abstract

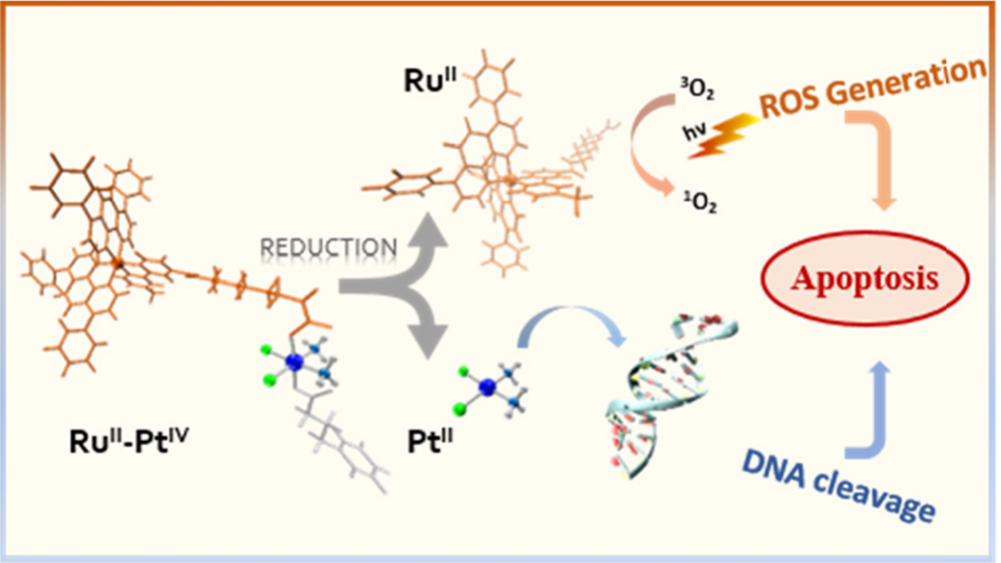

An in-depth computational study of the ability of a recently proposed multi-action Ru(II)–Pt(IV) conjugate to act as a photosensitizer in photodynamic therapy (PDT) and chemotherapeutic drugs is presented here. The investigated complex is characterized by a polypyridyl Ru(II) chromophore linked to a Pt(IV) complex that, acting as a prodrug, should be activated by reduction releasing the Ru-based chromophore that can absorb light of proper wavelength to be used in PDT. The reaction mechanism for active species formation has been fully elucidated by means of density functional theory and its time-dependent extension. The reduction mechanism, assisted by ascorbate, of the Pt(IV) prodrug to the Pt(II) active species has been explored, taking into consideration all the possible modes of attack of the reductant for releasing the axial ligands and affording active cisplatin. Given the similarity in the photophysical properties of the chromophore linked or not to the Pt(IV) complex, both the Ru(II)–Pt(IV) conjugate precursor and the Ru(II) chromophore should be able to act as PDT photosensitizers according to type I and type II photoprocesses. In particular, they are able to generate singlet oxygen cytotoxic species as well as auto-ionize to form highly reactive O_2_^–•^ species.

## Abstract 2

A computational study on the ability of a multi-action Ru(II)−Pt(IV) conjugate to act as a photosensitizer in photodynamic therapy (PDT) and chemotherapeutic drugs is presented here. The reduction mechanism of the Pt(IV) complex along with the photophysical properties of both the prodrug Ru(II)−Pt(IV) conjugate and Ru(II) complex is provided. The mechanism of action has been fully elucidated by means of density functional theory and its time-dependent extension.

## Introduction

Combining therapeutic agents is a strategy already used in clinical practices to hit multiple targets, maximize effectiveness, and overcome treatment resistance employing drugs with known activity that can act simultaneously with different mechanisms of action. Because of the urgency of developing new antineoplastic agents, multi-action drug design is becoming a pivotal research line in the fight against cancer.^1^

It is well known that platinum(II) complexes, with general structure [Pt(X)_2_(L)_2_], are the most effective anticancer agents currently used in a lot of chemotherapeutic regimens administered in clinical enviroments,^2−5^ However, they appear to be toxic because of their chemical reactivity and instability.^6^ Moreover, the bioavailability is limited, and oral administration is precluded because a large amount of Pt(II) drugs is lost in bloodstream before arriving at the ultimate target. In order to overcome the drawbacks of Pt(II) complexes, Pt(IV) complexes have been designed and synthesized as prodrugs.^7−9^ They are more kinetically inert^10^ and, as a consequence, can be even orally administered and should exhibit reduced side effects. Moreover, Pt(IV) prodrugs require to be activated inside the cells by biological reducing agents that allow square planar active Pt(II) species to be formed by the elimination of axial ligands.^11^ Among Pt(IV) complexes taken recently into consideration for this purpose, a cisplatin derivative, with two axial phenylbutyrate (PB) ligands, has been shown to be a potent cytotoxic agent against different cancer cell lines, even more potent than cisplatin or its Pt(IV) derivatives tethered to conventional axial ligands like hydroxido or acetato ligands.^12,13^ Because the rate of reduction is one of the most important parameters determining the efficacy of the Pt(IV) complexes as anticancer agents, the knowledge of the mode of activation is of pivotal importance. Our group has largely explored the mechanism of reduction of Pt(IV) complexes, in particular, using the monoanionic form of ascorbic acid (AscH^–^)^14,15^ that is the most abundant form at physiological pH (pKa ca. 3.8), as a reducing agent together with l-Cysteine (Cys), as a model of sulfur-containing bioreductants.

**Scheme 1:**
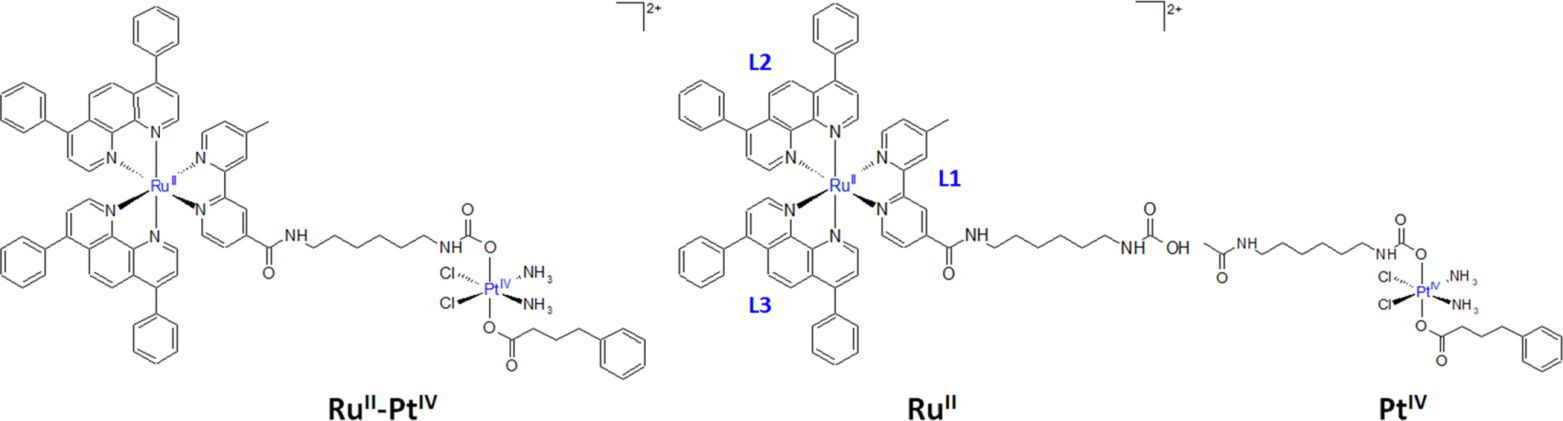
Structure of the Conjugate **Ru^II^**–**Pt^IV^**, the Photosensitizer **Ru^II^** Complex, and the Cisplatin Prodrug **Pt^IV^**

Besides conventional chemotherapy, in recent years, a great deal of attention has been devoted to a promising alternative therapeutic strategy that offers advantages compared to conventional treatments,^16,17^ that is, photodynamic therapy (PDT). The attractiveness of PDT relies on the use of safe doses of light and nontoxic concentrations of a chromophore, known as photosensitizer (PS), whose combined action induces cytotoxic and immunologic responses. Ideally, the PS confined in the target tissues, once activated by light, triggers a series of photochemical reactions that ultimately lead to selective destruction of diseased cells, minimizing off-target damage to the surrounding healthy tissues. In the framework of light-triggered therapeutic treatments, ruthenium complexes have emerged as a new generation of metal-containing antitumor drugs.^18^ Among the Ru-based complexes that have reached clinical trials,^19^ octahedral Ru(II) complex TLD1433 is the only photosensitizer candidate in PDT^20^ for the treatment of nonmuscle invasive bladder cancer. Because of intensive search for more active Ru photosensitizers, numerous complexes potentially active in DNA photocleavage and ^1^O_2_ generation have been synthesized. Among these, Ru(II) complexes bearing pyridyl ligands have been proposed as effective candidates for these strategies.^21−23^ A recent work reports the synthesis of a novel dual-action **Ru^II^**–**Pt^IV^** conjugate complex (Scheme 1), which combines the antineoplastic activity of platinum complexes with light-mediated action of Ru complexes to exert the ultimate cytotoxic effect.^24^ The authors have found the bimetallic conjugate to have a multitarget and multi-action effect. Indeed, the Pt(II) complex is released directly in the cell by the reduction of the corresponding Pt(IV) precursor, and its binding to nuclear DNA is favored by 4-phenylbutyrate (PB) action as a histone deacetylase (HDAH) inhibitor. At the same time, the Ru(II) complex that is accumulated at the Golgi apparatus catalytically generates singlet oxygen when irradiated with light of wavelength going from 480 nm up to 595 nm.^24^

Motivated by the promising results of the tests in vitro, we have undertaken a density functional theory (DFT) study to elucidate the mechanisms through which the proposed **Ru^II^**–**Pt^IV^** assembly can exert a multi-action effect. For this purpose, the reduction mechanism assisted by ascorbate of the **Pt^IV^** complex (Scheme 1), that allows the simultaneous release of cisplatin and PhB and Ru^II^ polypyridyl complex, has been explored in detail. On the other hand, time-dependent (TD) DFT has been used to investigate the photophysical properties of the **Ru^II^** photosensitizer and the whole **Ru^II^**–**Pt^IV^** conjugate in order to identify the most probable pathways for its photodynamic action. The outcomes presented here evidence that the cytotoxic action of the released active Pt(II) complex is reinforced by the photosensitizing activity of the Ru(II) chromophore that is not significantly influenced by the presence of the Pt(IV) unit.

## Computational Details

Quantum mechanical calculations have been performed employing the Gaussian 09 package^25^ at the restricted DFT (RDFT) level. The hybrid B3LYP functional,^26,27^ coupled with the D3 Grimme’s dispersion correction for nonbonding interactions,^28^ has been used for exploring both the reduction mechanism of the **Pt^IV^** complex and the ^1^O_2_ photosensitization **Ru^II^** and **Ru^II^**–**Pt^IV^** complexes. Optimizations have been performed using the cc-pVTZ basis set for all atoms,^29^ including Pt^30^ and Ru^31^ centers, for which the associated pseudo-potential has been employed. The spin multiplicity of all the examined complexes has been verified to be a singlet. Frequency calculations at the same level of theory have been performed to both calculate zero-point energy corrections and to identify local minima and transition states by the number of imaginary frequencies, 0 or 1, respectively. The located transition states have been checked to be properly connected to the corresponding minima by intrinsic reaction coordinate (IRC) analysis and to confirm that the vibrational mode associated with the imaginary frequency corresponds to the correct movement of the involved atoms.

The final energies have been obtained by performing single point calculations including solvent effects by means of the Solvation Model based on Density (SMD) with a dielectric constant of 78.3 for water solvent as implemented in Gaussian 09.^32^

Absorption spectra have been obtained as vertical electronic excitations from the optimized ground-state structure within the TD-DFT response theory by performing 150 electronic excitations. While B3LYP has been used for the optimizations in implicit water solvent as described above, a preliminary benchmark on the wavelength for the maximum absorption of the experimentally studied ruthenium polypyridyl complex^24^ (Figure S1) has been carried out in order to select the most suitable exchange and correlation functional. M06, M06L,^33^ B3LYP,^26,27^ PBE0,^34^ PBE,^35^ ϖB97XD,^36^ PW91,^37^ LC-wPBE,^38^ and CAM-B3LYP^26^ have been used for this purpose, and M06 has been selected. To establish the probability that a triplet state could be populated through ISC, spin-orbit matrix elements have been calculated using the SOC-TD-DFT approach implemented in Orca code.^39^ Relativistic corrections have been computed by the zeroth order regular approximation (ZORA) and its def2-SVP basis set at the ground-state optimized geometries. Accordingly, ZORA-DEF2-SVP and SARC-ZORA-SVP have been used for the main and metal atoms, respectively. Because of the use of the hybrid functional, M06, the RIJCOSX approximations was introduced to speed up the calculations’ time, as suggested in the ORCA manual. Spin-orbit couplings have been, thus, calculated using the following formula:
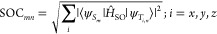


## Results and Discussion

### Reduction Mechanism

**Scheme 2:**
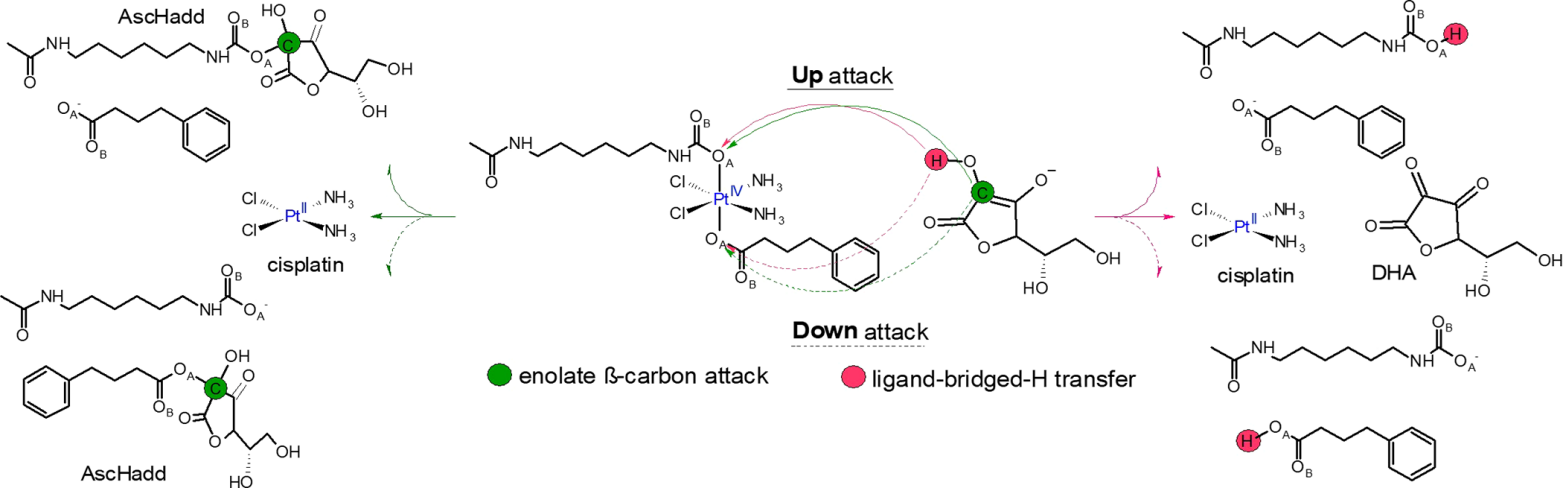
Investigated Ligand-Bridged H-Transfer (Pink) and Enolate β-Carbon Attack (Green) Reduction Mechanisms of **Pt^IV^** Assisted by AscH^–^ Occurring From Both Sides of the Complex and Named Up (Solid Lines) and Down (Dashed Lines)

Several mechanistic hypotheses have been proposed in the literature for the reduction of Pt(IV) complexes acting as prodrugs to afford the active Pt(II) species, whose occurrence can depend, for example, on the nature of the axial ligands as well as on the skeleton of the active square planar complex.^14,15^ The mechanisms for the two-electron transfer can be summarized in two main categories: outer- and inner-sphere mechanisms.^40−42^ The outer-sphere pathway does not entail that a contact is established between the reducing agent and the complex. A decomposition scheme involving the formation of a metastable six-coordinate Pt(III) intermediate has been proposed by Baik and co-workers^43^ to estimate both theoretically and experimentally the reduction potential.^14,15,44−46^ On the other hand, the inner-sphere mechanism can in principle follow three different pathways: (i) ligand-bridged, (ii) ligand-bridged-H transfer, and (iii) enolate β-carbon attack.^14,15,44,45,47^ The former involves the formation of a bridge between the metal center and the reducing agent facilitating the flow of the electrons toward the metal center through the axial ligand simultaneously promoting the *trans* ligand detachment. In the ligand-bridged-H transfer, the two-electron reduction takes place with the transfer of a H^–^ unit from the reducing agent to the axial ligand and the simultaneous formation of the oxidized form of AscH_2_, that is, dehydroascorbic acid (DHA). The last enolate β-carbon mechanism works when the reducing agent is the ascorbate and involves the nucleophilic attack of the ascorbate enolate β-carbon to the axial ligand (Scheme 2). The ligand-bridged-H transfer mechanism has been demonstrated to be the preferred one for hydroxido and carboxylato axial ligands.^14,15,44,45^

In order to investigate the reduction reaction, the **Ru^II^–Pt^IV^** conjugate complex has been modeled with the **Pt^IV^** complex reported in Scheme 1 because it has been clearly reported in the experimental work that the long aliphatic linker between Ru(II) and Pt(IV) moieties serves to make independent the activity of the two drugs.^24^ Thus, ligand-bridged-H transfer and enolate β-carbon attack in the presence of ascorbate as a model of the reducing agent have been explored by taking into consideration that the interaction can be established with both axial ligands, that are carbamate and PB. Then, the attacks have been here distinguished as Up and Down attacks, respectively as shown in Scheme 2. Moreover, the possibility that both oxygen atoms of the ligands, labeled O_A_ and O_B_, can undergo the attack has been taken into account.

**Figure 1:**
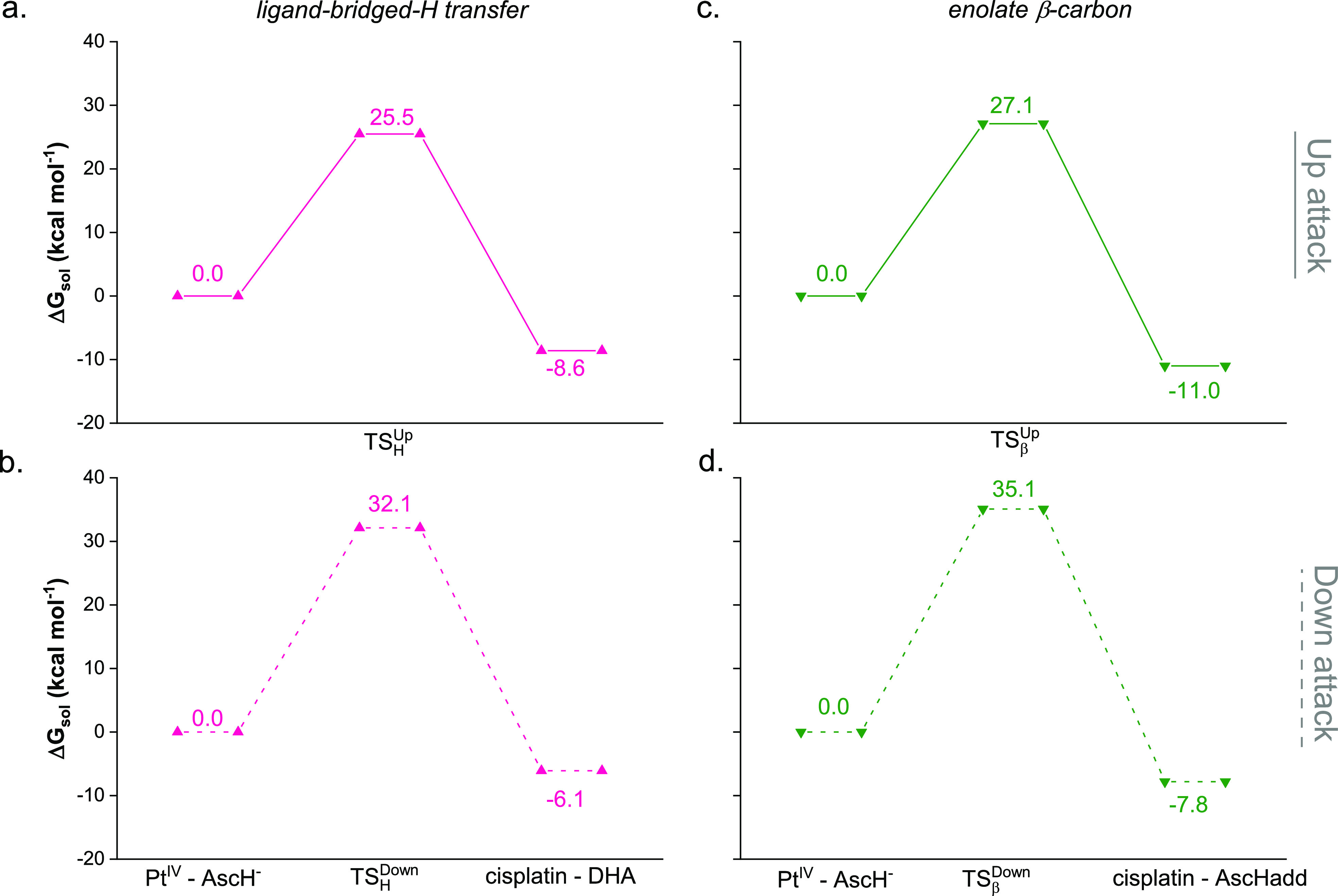
Free energy profiles describing the ligand-bridged-H transfer (pink lines) and enolate β-carbon (green lines) attack mechanisms for the reduction of complex **Pt^IV^** by ascorbate from the named Up (solid lines) or Down (dashed lines) sides of the complex. Energies (in kcal mol^–1^) are relative to the first adduct Pt^IV^-AscH^–^. Geometrical structures of the transition states are also included.

The reducing agents naturally present in the cells, that are l-ascorbic acid (AscH_2_) and l-glutathione, are considered the potential biological species responsible for the activation of Pt(IV) prodrugs by reduction.^40,48−51^ Accordingly, ascorbic acid has been used as the reducing agent and modeled in the most abundant form at physiological pH, the monoanionic AscH^–^, as sketched in Scheme 2. The outcomes of the attack on the oxygen atoms directly coordinated to the Pt center (named O_A_) are collected in Figure 1, while those coming from the attack on the other ligand oxygen atom (named O_B_) are reported in Figures S2 and S3. It is worth mentioning that we were unable to locate the stationary points accounting for the enolate β-carbon Up attack on the O_B_ atom despite the numerous attempts. The structural arrangements of all the stationary points intercepted along the free energy profiles of Figure 1 are provided in Figure S4. All the initial adducts formed by the **Pt^IV^** complex and the ascorbate are characterized by a H-bond established between the reducing agent OH group and the oxygen atom O_B_.

The reduction reaction, occurring along the ligand-bridged-H transfer pathway, takes place surmounting an energy barrier of 25.5 and 32.1 kcal mol^–1^ for the Up and Down attacks, respectively (Figure 1a, b, respectively) on the O_A_ atom. The H transfer from the AscH^–^ to the involved ligand and the release of the other axial ligand occur simultaneously for both attacks as evidenced by IRC calculations. Because no significant electronic differences on the two oxygen atoms coordinated to the Pt(IV) center have been observed, the computed difference in the height of the energy barrier could be imputable to the different arrangement of the entire carbamate ligand when the attack occurs from the side of the PB ligand (down attack). Indeed, in the involved transition state (TS_H_^Up^), the long chain establishes a series of stabilizing interactions with the other ligands missing in the other case, for which, instead, a substantial distortion of the octahedral structure around the metal can be observed (see the optimized structure of TS_H_^Down^).

As evidenced in Scheme 1, the formed products are cisplatin and the oxidized form of ascorbic acid, DHA, together with the protonated form, that is the corresponding acids, of one or the other axial ligand depending on the side, Up or Down, of attack. However, reaction is exergonic in both cases, 8.6 and 6.1 kcal mol^–1^ for the former and latter pathway, respectively.

The same reaction mechanism involving O_B_ results equally probable from the Up or Down sides, because an energy barrier of 29.4 and 30.6 is computed, respectively, and the product is more stable by 0.3 and 1.7 kcal mol^–1^, respectively, than the first adduct used as the reference. Comparing the energies put into play according to the H-transfer mechanism involving O_A_ or O_B_, the Up attack results the most probable one regardless of the oxygen atom undergoing the attack and, although the Down attack on the O_B_ is slightly favored than that on OA from a kinetic point of view, the exergonicity is always in favor of the O_A_ attacks.

Similarly, the enolate β-carbon attack is slightly more favorable when occurring on the ligand from the Up side (Figure 1c), requiring 27.1 kcal mol^–1^ to take place against 35.1 kcal mol^–1^ needed for the Down attack. Even here, the cisplatin formation is accompanied by the generation of an ascorbate with an additional carboxylate moiety on the enolate β-carbon (AscHadd), the results of which are exergonic by 11.0 and 7.8 kcal mol^–1^ for Up and Down attacks, respectively. Looking at the optimized structure of the involved transition states, it appears that the enolate β-carbon attack is accompanied by a strong distortion of the hexa-coordination of the metal because of the partial formation of a new C–O bond already in the transition state, especially in the case of the Up attack. Thus, the distortion of the first coordination shell around the platinum center should contribute to the higher energy barrier found in the enolate β-carbon mechanism. The slightly larger stability of the formed products (cisplatin-AscHadd) following such a mechanism if compared with the ligand-bridged-H transfer products (cisplatin-DHA) could be misleading because the described enolate β-carbon attack has to be followed by a second step to form the final product DHA. Such a step, investigated in detail for the asplatin complex,^45^ is reported to be the slowest step of the whole enolate β-carbon mechanism, and the final product is destabilized by about 10 kcal mol^–1^ with respect to the product preceding it.

The outcomes of our calculations confirm that the H-transfer mechanism is the preferred pathway for the **Pt^IV^** complex investigated here, as most cases reported in the literature.^14,15,44,45^ Moreover, the computed favored attack (Up) from the side of the carbamate group is consistent with the behavior observed by Zhu and co-workers, who studied the cytotoxicity and the reduction rate of a series of carbamate and carboxylate Pt(IV) drugs, finding that the former is faster reduced than the latter.^52^

To take into consideration the viability of the outer-sphere mechanism, the standard redox potential of the **Pt^IV^** complex has been calculated according to the Baik and coworker decomposition scheme,^43^ already successfully applied to other Pt(IV) complexes.^14,44−46^ The reduction potential is computed as the energy change that accompanies the one-electron transfer for the reduction of the six-coordinate **Pt^IV^** to a six-coordinate Pt(III) species named ΔG_sol_. Thus, the following equation has been used:
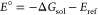
where *E*_ref_ is the absolute potential of the standard electrode used as a reference (SHE). The value of −0.31 eV computed for the reduction potential of the **Pt^IV^** complex, compared with the data reported in the literature for outer-sphere reductions,^14,44−46^ suggests that such a mechanism cannot be considered as viable in the present case.

Therefore, the electron transfer occurs, very likely, in an inner-sphere fashion through a ligand-bridged-H transfer mechanism, leading to cisplatin formation accompanied by the release of the **Ru^II^** photosensitizer and HDAC inhibitor PB. More importantly, the energies coming into play confirm that the reduction occurs already in dark once the conjugate **Ru^II^**–**Pt^IV^** enters the cell environment, and the released separate cisplatin and **Ru^II^** components can thus exert synergistically their antineoplastic activities. Thence, the photodynamic action of the **Ru^II^** component alone has been explored.

### Absorption Spectra

In order to accurately describe the photophysical features of the complexes under examination, a preliminary benchmark has been carried out on the absorption spectrum the ruthenium polypyridyl complex experimentally characterized in acetonitrile.^24^ For the selection of the most suitable protocol, attention has been focused on the reproduction of the wavelength for the maximum absorption of the low frequency band that is the most important parameter for PDT applications. Several exchange and correlation functionals have been taken into consideration, and the results are provided in Figure S4. From these data, the hybrid M06 functional emerges as a better choice to simulate the electronic spectrum of the Ru(II) complex experimentally recorded.^24^ Thus, M06 has been employed for the complete characterization of the absorption spectra of both **Ru^II^** and **Ru^II^–Pt^IV^** chromophores of Scheme 1, computed in the solvent water that better mimics the physiological environment. It is worth noting that **Ru^II^** considered here is exactly the complex resulting from the reduction process of the Pt(IV) complex to afford cisplatin. Thus, it bears the entire long chain that is detached from the Pt center as a consequence of reduction (see Scheme 1).

**Figure 2:**
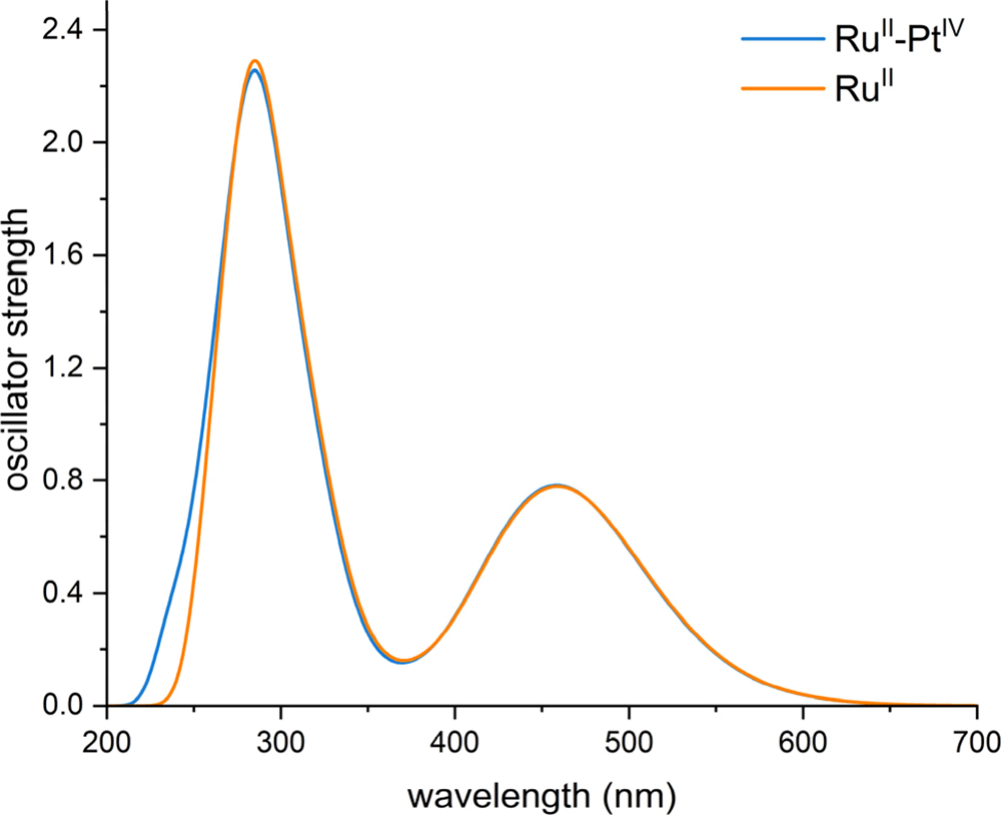
TD-DFT simulated absorption spectra of **Ru^II^** (orange) and **Ru^II^–Pt^IV^** (blue) complexes in the water environment.

The obtained spectra are both characterized essentially by two absorption bands, the most intense in the region 250–350 nm and a less intense band within 380 and 500 nm (Figure 2), according to the photophysical features experimentally found.^24^ Thus, appending the chromophore to the **Pt^IV^** moiety does not change significantly the shape of the absorption spectrum of the **Ru^II^** complex alone (see Figure 2), especially looking at the low frequency region pivotal for PDT application.

The lowest energy band, peaked at 469 nm for both the complexes, originates from different electronic transitions that start mainly from the highest occupied molecular orbitals (from HOMO(H) to H-2) mainly centered on the metal and terminate to unoccupied orbitals comprised between LUMO(L) and L+3 in **Ru^II^** and L+2 and L+5 in the conjugate **Ru^II^–Pt^IV^**, which are orbitals centered on the bipy ligands. Overall, the transition can be ascribed to a metal-to-ligand charge transfer (MLCT), where the CT occurs from the Ru(II) center to all the surrounding bipyridine ligands (see Tables S1 and S2 and Figures S5 and S6). In particular, the lowest electronic transition involves different MOs for the two complexes; it is almost purely H → L in **Ru^II^** and becomes H → L+2 in **Ru^II^–Pt^IV^**. It is computed with a very low oscillator strength at 548 and 545 nm, and the charge transfer essentially implicates the ligand named L1 in Scheme 1, connected to the Pt center in the **Ru^II^–Pt^IV^** conjugate. Although the convoluted band shape does not explicitly show the shoulder at higher energy experimentally found (438 nm), it is reasonable to hypothesize that the electronic transition computed at 438 and 437 nm for **Ru^II^** and **Ru^II^–Pt^IV^** complexes, respectively, is responsible for such a band. It is originated by the of H-2 → L+5 transition for the **Ru^II^–Pt^IV^** dyad and of H-2 → L+3 for the **Ru^II^** complex. However, the character of the band remains to be MLCT. The very similar behavior found for the two complexes clearly evidences the insensitiveness of the **Ru^II^** chromophore to the presence of the Pt(IV) unit. It can be ascribed to the nature of the MOs involved in such transitions that determine the shape of the natural transition orbital (NTO) plots reported in Figures S5 and S6 which show, indeed, that the Pt(IV) moiety is not involved at all.

Even the most intense band is originated by several electronic transitions that involve just the ligands of the chromophore, and thus, it is mainly of the type ligand-to-ligand charge transfer (LLCT), although slight participation to the band of a charge transfer from the ligand to the metal (LMCT) can be observed in both cases. As it usually occurs for the high frequency band, the involved electronic transitions start from the inner MOs (e.g., H-12, H-16) and arrive to higher energy unoccupied orbitals (e.g., L+9, L+12); see Tables S2 and S3 for further details.

### Photodynamic Action

**Figure 3:**
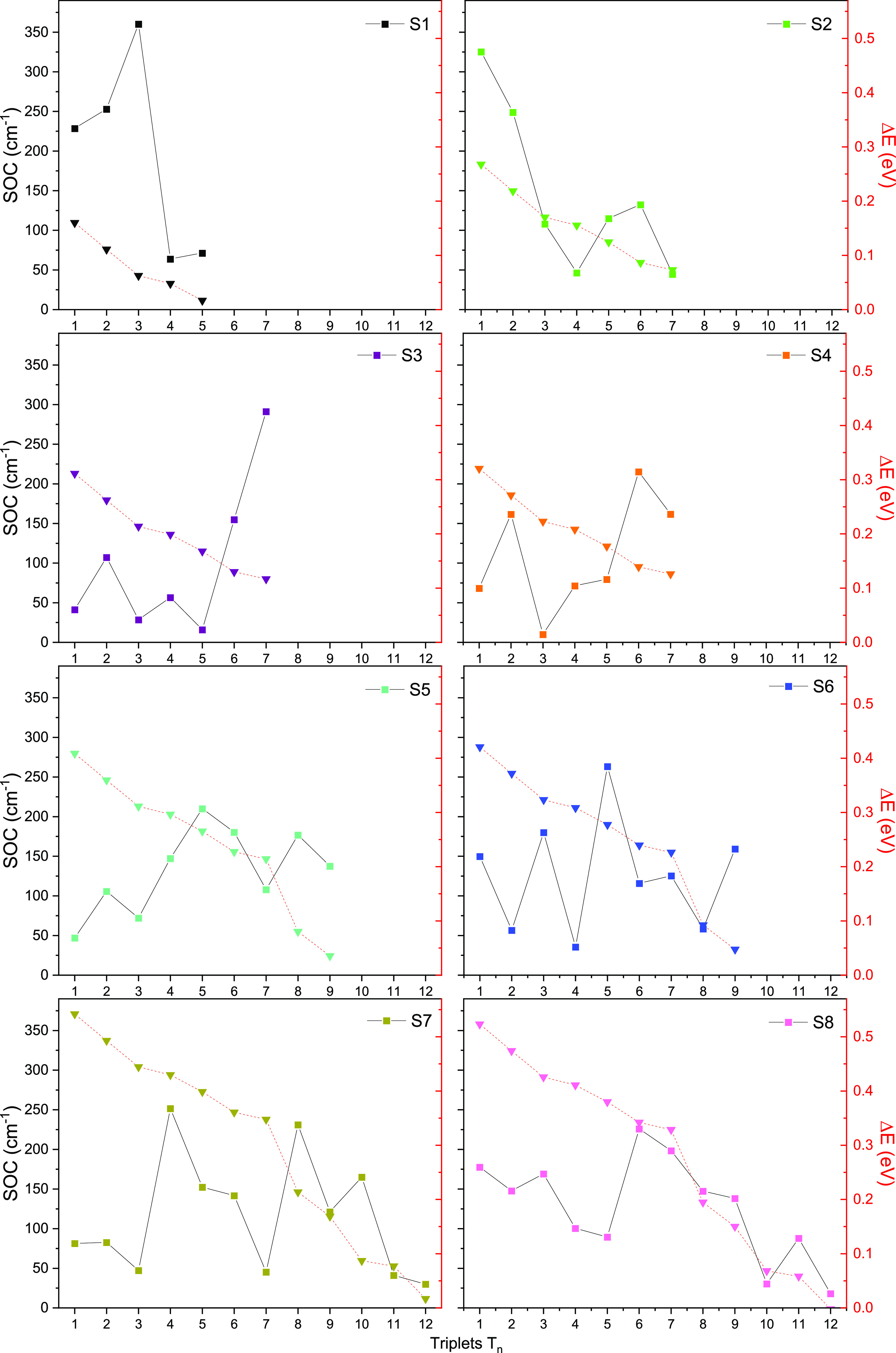
SOC values (cm^–1^) for the S_*m*_ → T_*n*_ (with *m* = 1–8 and *n* = 1–12) radiationless transitions and singlet–triplet energy gaps (eV) computed for the **Ru^II^** complex.

The chromophore can accomplish its photodynamic action by triggering two different photochemical processes, namely, type I and type II photoreactions. Both pathways require that the photosensitizer possesses specific properties to be used in therapeutic practice, the most important of which is surely the absorption wavelength falling within the therapeutic window. As reported in the previous section, as the maximum absorption of both the **Ru^II^–Pt^IV^** conjugate and the **Ru^II^** complex falls in the down region of the therapeutic window, they can in principle be able to act as drugs in PDT. The other key characteristic is the existence of a triplet state of proper energy because once the photon is absorbed, an ISC process has to occur from the singlet state to a triplet one. Therefore, a considerable spin–orbit coupling from the excited singlet to triplet electronic states is required to trigger either the energy transfer from the complex to molecular oxygen ^3^O_2_ (type II) or the electron transfer to promote autoionization reactions (type I). In type II photoreactions, the photosensitizer triplet energy has to be greater than the energy of the^3^Σ_g_^–^–^1^Δ_g_ gap of O_2_. It should be pointed out that the amount of energy required to generate the singlet oxygen has been computed to be 0.90 eV, in good agreement with the experimental value of 0.98 eV.^53^ Both the **Ru^II^** complex and its conjugate **Ru^II^–Pt^IV^** have several triplet states with energy higher than 0.90 eV and lying below the lowest energy singlet states belonging to the first absorption band. Thus, assuming that the absorption phenomenon should lead to the population of the state with the highest oscillator strength generating the first band located at around 2.6 eV in both complexes (see Tables S1 and S2), all the triplet states lying below such state have been taken into consideration to explore all the possible singlet deactivation pathways through the ISC process (Tables S3 and S4). According to the Fermi golden rule, in the framework of Marcus–Levich–Jortner theory,^54,55^ the ISC kinetics essentially depends on two main parameters:^56^ the spin-orbit couplings (SOC) and the singlet-triplet splitting energies (Δ*E*) between the coupled states. Both these parameters have been computed for the **Ru^II^** complex (Table S5) and for the pro-drug **Ru^II^–Pt^IV^** (Table S6). However, in order to make easier the selection of the most probable ISC channels, a plot of the values of both SOC and Δ*E* values calculated for the **Ru^II^** complex has been reported in Figure 3.

**Figure 4:**
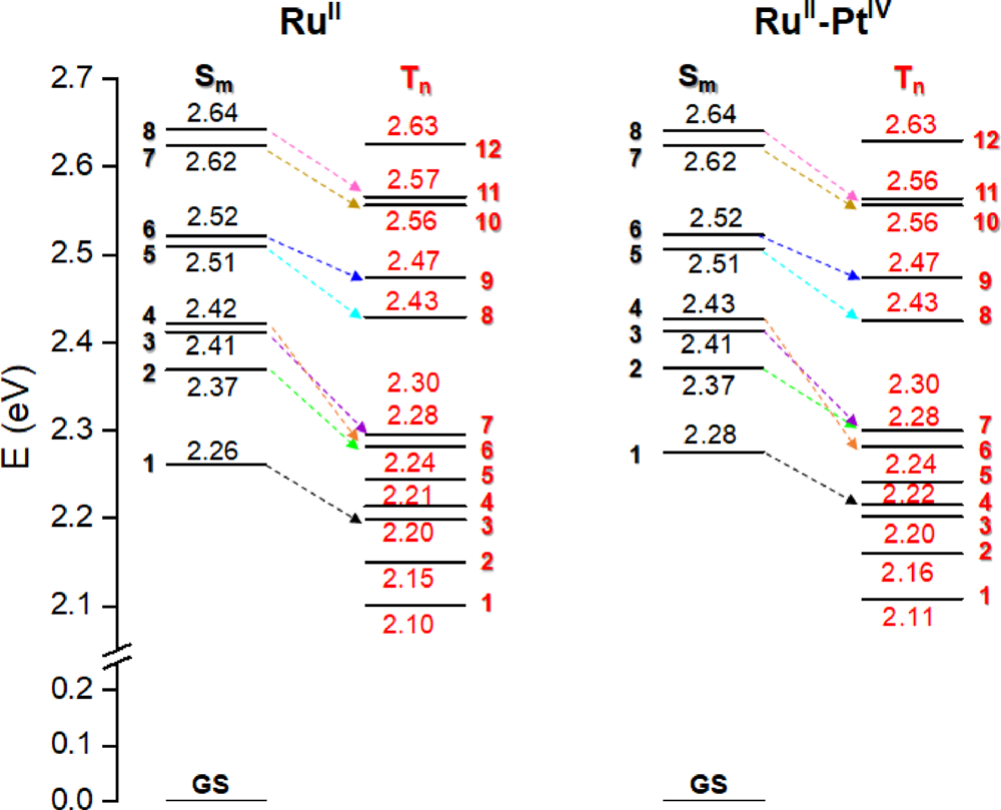
Energy diagram of the low-lying excited singlet (black) and triplet (red) states of the **Ru^II^** complex and its precursor **Ru^II^–Pt^IV^** computed with respect to the ground-state zero energy. Dashed colored arrows indicate the most probable coupling of each singlet state with one of the triplet state lying below as resulted from the data reported in Figure 3.

Data collected in Tables S5 and S6 confirm the similar behavior of the photosensitizer **Ru^II^** and its precursor **Ru^II^–Pt^IV^**, with regard to both excitation energies, for singlet and triplet states, and, to some extent, the SOC values. Moreover, all the calculated SOCs are large and surely support the triplet state population and, then, singlet oxygen production that can be triggered through radiationless transitions between all the considered singlet and triplet states. However, looking at both SOC and Δ*E* values, for all the S_*m*_ (with *m* = 1–8) singlet states potentially involved in the ISC process (see Tables S1 and S2), only the singlet deactivation channels considered more plausible have been highlighted in Figure 4. In such selection, all the energy transfer occurring among states separated by a too large gap have been discarded, despite having high SOC values.

Therefore, looking at Figure 3, the fastest radiationless transition should involve one of the bright singlet states among S_7_ (that mostly contributes to the first absorption band), S_6_, S_5_, and S_1_ because they exhibit the highest SOCs (between 150 and 200 cm^–1^ for all, except S_1_ for which the coupling with T_3_ corresponds to a value of 360 cm^–1^) among states closer in energy (energy gap less than 0.1 eV).

Considering the most probable transitions involving the states explicitly highlighted in Figure 4, the associated NTOs (reported in Figures S5 and S7) evidence that the coupling can occur between states with different character. For example, in the case of S_7_ → T_10_, certainly MLCT occurs in both states, but in the case of T10, a charge transfer between ligands contributes to the generated state, L_1_L_2,3_CT, changing the shape of its NTOs. However, according to the El-Sayed rules, a considerable SOC value has been steadily computed when a change of the orbital type accompanies coupling, see, for example, S_6_–T_9_, S_5_–T_8_, and S_1_–T_3_ couplings.

As already stated for the absorption properties, the ability of the **Ru^II^** complex to trigger type II photoreactions aimed at generating the cytotoxic agent is not significantly influenced by the presence of the bound Pt(IV) moiety, which does not play any role in **Ru^II^–Pt^IV^** transitions, as shown by the nature of the NTOs.

The alternative feasible photodynamic mechanism, type I process, is based on the reduction of the photosensitizer in its T1 state by an organic substrate and involves the transfer of an electron or an H atom to a biomolecule or to the sensitizer itself. The formed radical can thus react with molecular oxygen ^3^O_2_ to generate the superoxide oxygen radical species O_2_^–•^ and other reactive oxygen species (ROS) appointed to destroy the cells in which they have been produced. The viability of photoinduced electron transfer reactions can be established computing the vertical electron affinity (VEA) and ionization potentials (VIP) for complexes and molecular oxygen.^57^ These values have been calculated in water and are collected in Table 1.

**Table 1:** VEA And VIP Values (eV) Computed in Water at the M06/cc-pVTZ Level of Theory for ^3^O_2_^a^ and **Ru^II^** and **Ru^II^–Pt^IV^** Complexes, in Their Singlet and Triplet Excited States

	VEA	VIP	VEA(T1)	VIP(T1)
**Ru^II^**	–2.78	5.10	–4.70	3.18
**Ru^II^–Pt^IV^**	–2.77	5.11	–5.03	2.84

a VEA O_2_ = −2.94 eV computed at the M06/cc-pVTZ level in water.

The pathway through which the metal complex can produce O_2_^–•^ reacting with molecular oxygen can occur either by direct electron transfer or passing from the autoionization reactions, according to which the reduction of the T1 state of the photosensitizer is realized by the neighboring S0 or T1 state of the photosensitizer itself. Such reactions generally lead to the photosensitizer radical anion or radical cation formation being neutral in most of molecules proposed for PDT applications. In the case of Ru-based complexes, generally indicated as [Ru]^2+^, having total charge +2, the autoionization reactions can be schematized as follows:

(a) ^3^[Ru]^2+^ + ^1^[Ru]^2+^ → [Ru]^3+•^ + [Ru]^+•^
(b) ^3^[Ru]^2+^ + ^3^[Ru]^2+^ → [Ru]^3+•^ + [Ru]^+•^

The occurrence of the first process can be established by looking at the VEA and VIP values of metal complexes in their triplet and singlet states, respectively, whose sum must be less than zero. Thus, reaction (a) is not feasible for both complexes, VIP being greater than VEA, and then, the sum leads to positive values (0.41 and 0.07 eV for **Ru^II^** and **Ru^II^–Pt^IV^** complexes, respectively).

However, the triplet state of Ru-based complexes can be reduced through auto-ionization reaction (b) by the neighboring species of the same nature, ^3^[Ru]^2+^; indeed, the absolute value of the vertical electron affinity of the triplet states is greater than the ionization potential of **Ru^II^** and **Ru^II^–Pt^IV^** complexes of 1.51 and 2.19 eV, respectively; therefore, the sum of VEA and VIP of the complexes in the triplet states returns out negative.

As stated above, the superoxide anion O_2_^–•^ formation can be realized by direct electron transfer from the photosensitizer (in its triplet state) to molecular oxygen or through electron transfer from the reduced form of metal complexes to ^3^O_2_ as illustrated below:

(c) ^3^[Ru]^2+^ + ^3^O_2_ → [Ru]^3+•^ + O_2_^–•^
(d) [Ru]^+•^ + ^3^O_2_ → ^1^[Ru]^2+^ + O_2_^–•^

To establish if Ru-based complexes can follow the direct pathway (c), it should be verified that the sum of molecular oxygen VEA and triplet photosensitizer VIP is negative, while for the occurrence of the last process, (d) is the sum of the VEA of molecular oxygen and the VIP of [Ru]^+•^ (which is equal to minus the VEA of [Ru]^2+^) that should lead to the same result. The former pathway is accessible only for the **Ru^II^–Pt^IV^** complex because the energy of the process for **Ru^II^** is unfavorable (0.24 eV). The negative values of VEA computed for both complexes evidence a good propensity of the photosensitizers to receive an electron; thus, the feasibility of pathway (d) is rather evident from the energy of the process equal to −0.17 and −0.18 eV for **Ru^II^** and **Ru^II^–Pt^IV^** complexes, respectively. Therefore, it is reasonable that the highly reactive O_2_^–•^ species can be obtained by autoionization of [Ru]^2+^ complexes in their triplet state through photoreaction (b) and subsequent electron transfer to molecular oxygen, pathway (d).

## Conclusions

The mechanism of action of a Pt(IV) prodrug, **Ru^II^–Pt^IV^**, combining the cytotoxic properties of the cisplatin complex and the photosensitizing activity of the polypyridyl Ru(II) chromophore, **Ru^II^**, that are released when the complex is reduced in the cellular environment, has been computationally examined. The photophysical properties of **Ru^II^** have been compared with those of **Ru^II^–Pt^IV^** aiming at verifying whether both can be used as photosensitizers, and the properties of the Ru(II) complex are influenced by the presence of the bound Pt(IV) complex.

The mechanism by which the Pt(IV) complex is reduced has been examined using the monodeprotonated form of ascorbic acid as a model of the reducing agent revealing that the inner-sphere mechanism, labeled bridged-H-transfer, is the preferred one when the oxygen atom directly bound to the metal center of the monosuccinimidylcarbonate ligand is involved.

Both basic pathways, type I and type II, by which the combination of a metal complex as PS, light, and O_2_ results in photosensitized cell death, have been explored. **Ru^II^** fulfills all the requirements to act as a drug in PDT even if the maximum absorption falls in the down region of the therapeutic window. Moreover, outcomes evidence that the action of the Ru(II) chromophore is preferentially explicated through type II than type I photoprocess and is not significantly influenced by the presence of the bound Pt(IV), which does not play any role in **Ru^II^–Pt^IV^** transitions, as shown by the nature of the involved NTOs. This work provides an insight into the mechanism of action of a novel multi-action Ru(II)–Pt(IV) conjugate, and the results indicate that the efficacy of released cisplatin anticancer drug can be enhanced by the incorporation of a chromophore acting as a photosensitizer.

## Supporting Information Available

Benchmark of exchange and correlation functionals on the reproduction of the wavelength for the maximum absorption of ruthenium polypyridyl complex reported; free energy profiles describing the ligand-bridged-H transfer and enolate β-carbon attack mechanisms on O_B_ for the reduction of complex Pt^IV^ by ascorbate from the named Up or Down sides of the complex; optimized structure of the stationary points intercepted along all the investigated potential energy surfaces for reduction mechanisms occurring for the attack to O_B_ and O_A_; excitation energies; absorption wavelength; oscillator strength; MO contribution for **Ru^II^** and **Ru^II^–Pt^IV^** complexes; vertical electronic excitations of **Ru^II^** and **Ru^II^–Pt^IV^** complexes and NTOs for the excited states with oscillator strength greater than 0.1; triplet states excitation energies; MO contribution and theoretical assigned character for **Ru^II^** and **Ru^II^–Pt^IV^** complexes; SOC values for the S_*n*_ → T_*m*_ radiationless transitions and singlet–triplet energy gaps (eV) computed for **Ru^II^** and **Ru^II^–Pt^IV^** complexes; and NTOs for the excited triplet states T_*n*_ of **Ru^II^** and **Ru^II^–Pt^IV^** complexes (PDF)
