# Supplementary material for: Computational Exploration of the Synergistic Anticancer Effect of a Multi-Action Ru(II)–Pt(IV) Conjugate
Source: Inorg Chem. 2022 Jul 28;61(32):12903–12. doi: 10.1021/acs.inorgchem.2c02223 (PMC9382638; doi:10.1021/acs.inorgchem.2c02223)
Supplement: Supplementary file 1 — ic2c02223_si_001.pdf [file ic2c02223_si_001.pdf]

Supporting Information

Computational exploration of the synergistic anticancer effect of a multi-action Ru(II)-Pt(IV) conjugate.

Stefano Scoditti,[1] Gloria Mazzone,[1,*] Nico Sanna[2] and Emilia Sicilia[1]

[1] Dipartimento di Chimica e Tecnologie Chimiche, Università della Calabria, 87036 Rende, CS, Italy.

[2] Department for Innovation in Biology Agro-Food and Forest Systems (DIBAF), University of Tuscia, Largo dell'Università snc, 01100 Viterbo, Italy.

Table of Contents

- **Figure S1:** Benchmark of exchange and correlation functionals on the reproduction of the wavelength for the maximum absorption of ruthenium polypyridyl complex reported. **S-2**
- **Figure S2:** Free energy profiles describing the ligand-bridged-H transfer (pink lines) and enolate β-carbon (green lines) attack mechanisms on O_B for the reduction of complex Pt^{IV} by ascorbate from the named Up (solid lines) or Down (dashed lines) sides of the complex. **S-3**
- **Figure S3:** Optimized structure of the stationary points intercepted along all the investigated potential energy surfaces for reduction mechanisms occurring for the attack to O_B. **S-4**
- **Figure S4:** Optimized structure of the stationary points intercepted along all the investigated potential energy surfaces for reduction mechanisms occurring for the attack to O_A. **S-5**
- **Table S1:** Excitation energies (ΔE, eV), absorption wavelength (λ, nm), oscillator strength (f), MO contribution (%) for **Ru^{II}** complex. **S-6**
- **Table S2:** Excitation energies (ΔE, eV), absorption wavelength (λ, nm), oscillator strength (f), MO contribution (%) for **Ru^{II}-Pt^{IV}** assembly. **S-7**
- **Figure S5:** Vertical electronic excitations of **Ru^{II}** complex and NTOs for the excited states with oscillator strength greater than 0.1. **S-8**
- **Figure S6:** Vertical electronic excitations of **Ru^{II}-Pt^{IV}** dyad and NTOs for the excited states with oscillator strength greater than 0.1. **S-13**
- **Table S3:** Triplet states excitation energies (eV), MO contribution (%) and theoretical assigned character for **Ru^{II}** complex. **S-18**
- **Table S4:** Triplet states excitation energies eV), MO contribution (%) and theoretical assigned character for **Ru^{II}-Pt^{IV}** assembly. **S-19**
- **Table S5**. SOC values (cm^{-1}) for the $S_n \rightarrow T_m$ (with n = 1-8) radiationless transitions and singlet–triplet energy gaps (eV) computed for **Ru^{II}** complex. **S-20**
- **Table S6**. SOC values (cm^{-1}) for the $S_n \rightarrow T_m$ (with n = 1-8) radiationless transitions and singlet–triplet energy gaps (eV) computed for the pro-drug **Ru^{II}-Pt^{IV}**. **S-21**
- **Figure S7:** NTOs for the excited triplet states T_n (n = 1-12) of **Ru^{II}** complex. **S-22**
- **Figure S8:** NTOs for the excited triplet states T_n (n = 1-12) of **Ru^{II}-Pt^{IV}** complex. **S-25**

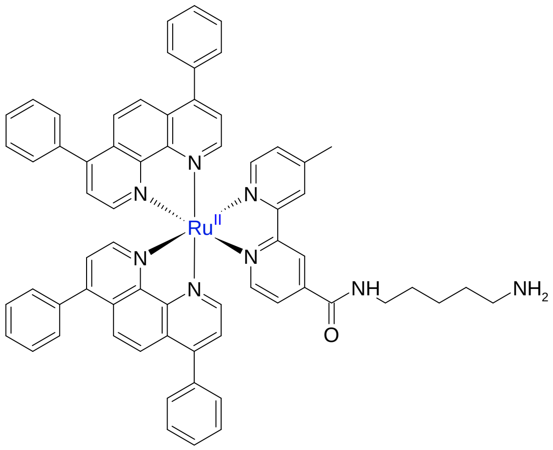

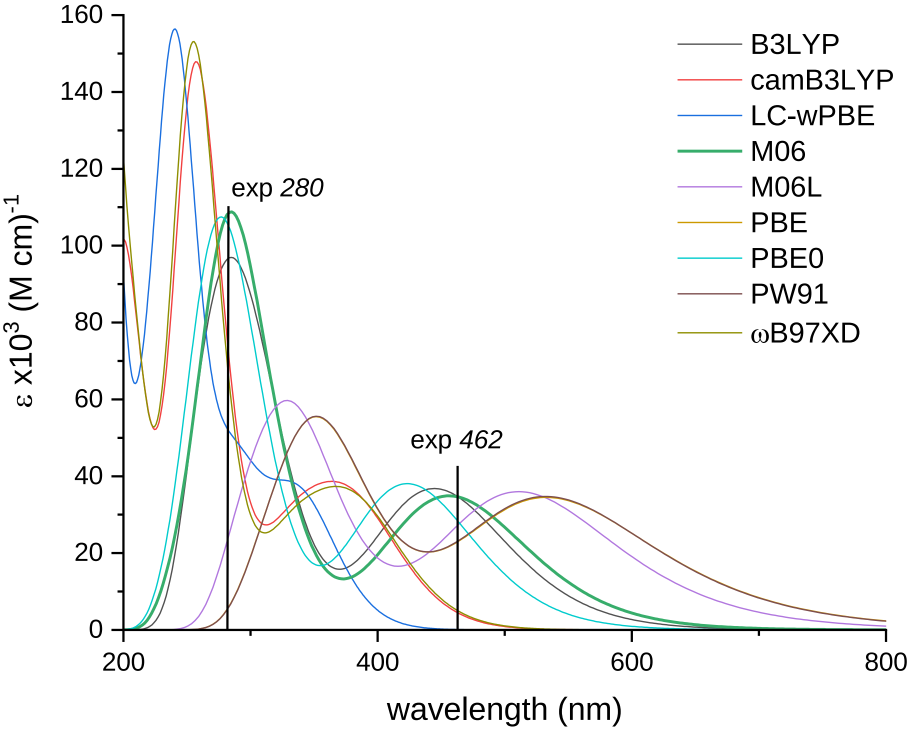

Figure S1: Benchmark of exchange and correlation functionals on the reproduction of the main wavelengths absorption of ruthenium polypyridyl complex reported. Experimental values are taken from Karges, J.; Yempala, T.; Tharaud, M.; Gibson, D.; Gasser, G. *Angew. Chemie - Int. Ed.* **2020**, *59* (18), 7069–7075

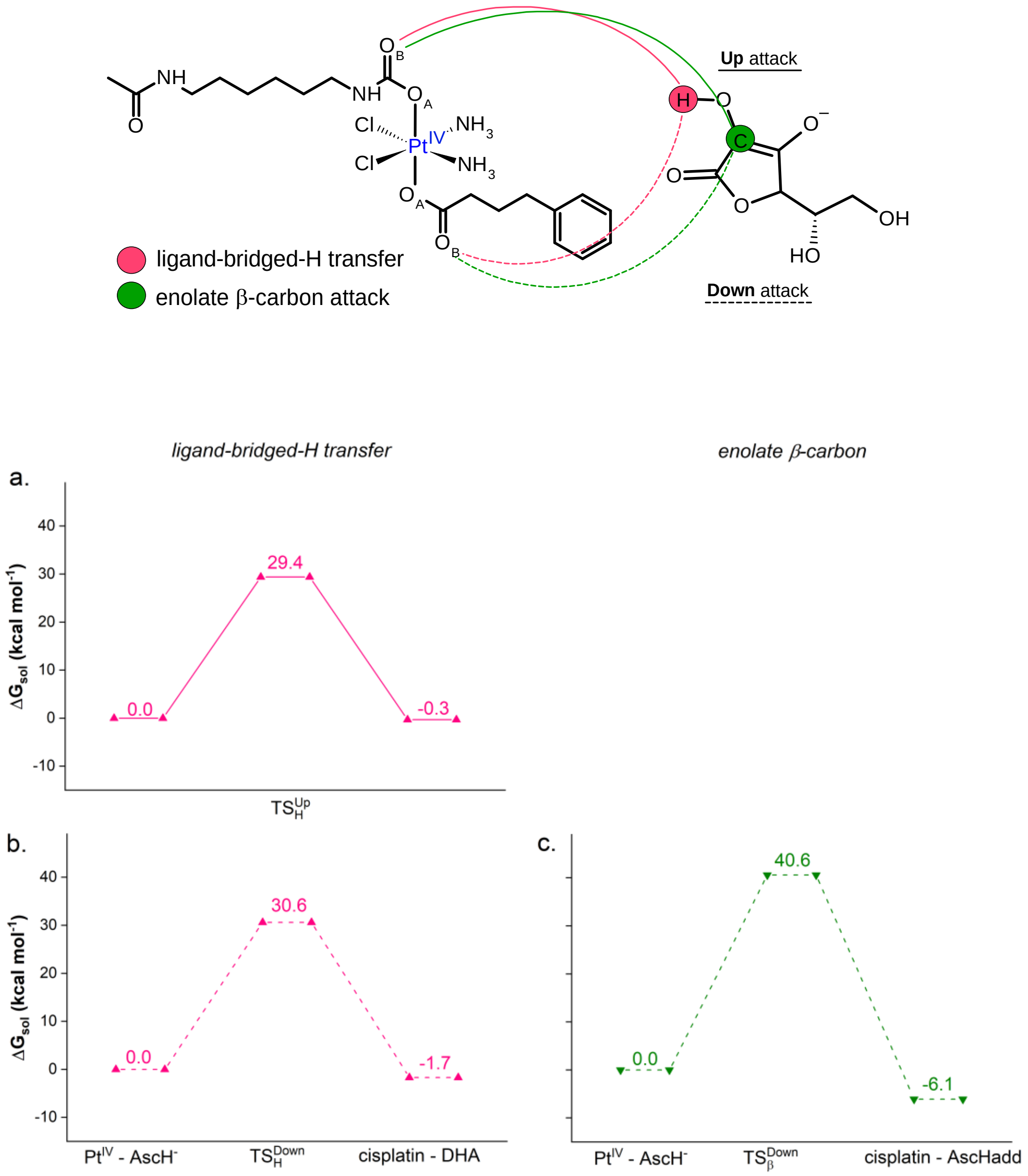

Figure S2. Scheme and Free energy profiles of ligand-bridged-H transfer (pink lines) and enolate β-carbon (green lines) mechanisms for the $AscH^-$ attack form the Up (solid lines) and the Down (dashed lines) sides of the **Pt^{IV}** complex on O_B atom.

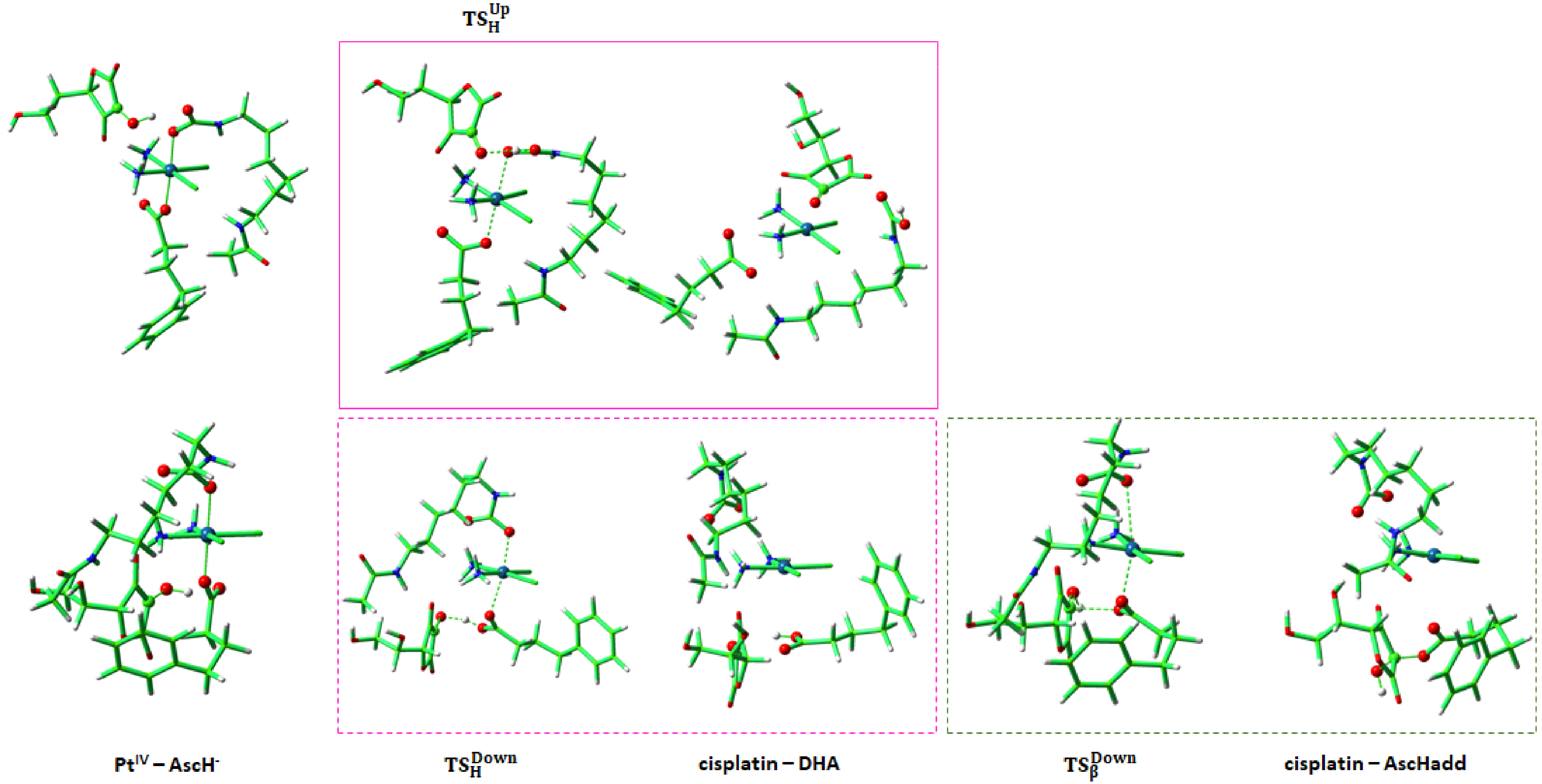

Figure S3: Optimized structure of the stationary points intercepted along all the investigated potential energy surfaces for reduction mechanisms occurring for the attack to O_B.

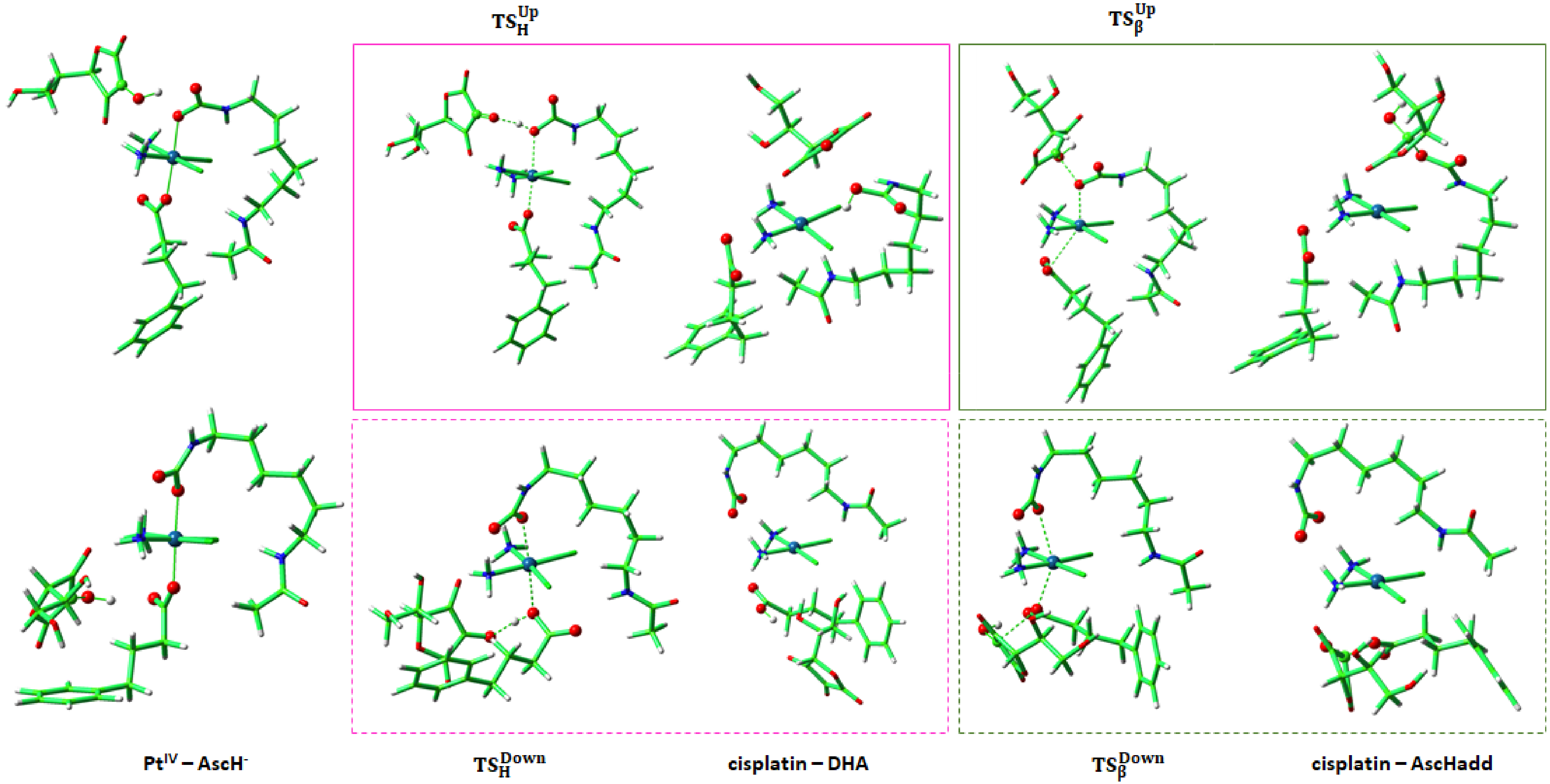

Figure S4: Optimized structure of the stationary points intercepted along all the investigated potential energy surfaces for reduction mechanisms occurring for the attack to O_A.

Table S1: Excitation energies (ΔE, eV), absorption wavelength (λ, nm), oscillator strength (f), MO contribution (%) for **Ru^{II}** complex.

Band	ΔE	λ	*f*	MO contribution	Theoretical Assignment
I	2.26	548	0.004	H → L 73%	**MLCT**
	2.37	523	0.009	H-1 → L 72%	
	2.41	514	0.007	H → L +1 69%	
	2.42	512	0.001	H → L +2 80%	
	2.51	494	0.107	H-2 → L 49%	
				H-1 → L+1 21%	
	2.52	492	0.097	H-2 → L 43%	
				H-1 → L+1 21%	
	2.62	472	0.099	H-2 → L+1 49%	
				H-1 → L+2 34%	
	2.64	469	0.192	H-2 → L+2 41%	
				H-1 → L+1 30%	
	2.83	438	0.150	H-2 → L+3 73%	
II	3.91	317	0.267	H-3 → L+3 39%	**LLCT**
	3.92	316	0.177	H-3 → L+4 28%	
				H-4 → L+3 24%	
	4.17	298	0.163	H-1 → L+8 28%	
				H-7 → L 25%	
	4.19	296	0.222	H-7 → L 31%	
	4.27	290	0.252	H-5 → L+3 26%	
				H-6 → L+4 15%	
	4.32	287	0.164	H-6 → L 23%	
				H-6 → L+1 10%	
	4.33	286	0.139	H-1 → L+9 38%	
	4.37	284	0.179	H-1 → L+10 21%	
				H-6 → L 19%	
				H-1 → L+9 13%	
	4.39	283	0.137	H → L+10 47%	
	4.43	280	0.146	H-16 → L 24%	
				H-6 → L 12%	
	4.50	275	0.230	H-5 → L+2 27%	
				H-7 → L+1 14%	
	4.71	263	0.226	H-12 → L+4 18%	
				H-13 → L+3 17%	
	4.93	251	0.127	H-7 → L+5 47%	

a. only vertical transitions with oscillator strength greater than 0.1 are reported, with the exception of the most red-shifted transition.

Table S2: Excitation energies (ΔE, eV), absorption wavelength (λ, nm), oscillator strength (f), MO contribution (%) for **Ru^{II}-Pt^{IV}** assembly.[a]

Band	ΔE	λ	*f*	MO contribution	Theoretical Assignment
I	2.27	545	0.004	H → L+2 68%	**MLCT**
				H → L+3 23%	
	2.37	523	0.010	H-1 → L+2 65%	
				H-1 → L+3 21%	
	2.41	514	0.006	H → L+3 63%	
				H → L+2 24%	
	2.43	511	0.001	H → L+4 86%	
	2.51	495	0.089	H-2 → L+2 33%	
				H-1 → L+3 27%	
	2.52	491	0.118	H-2 → L+2 60%	
	2.62	472	0.113	H-2 → L+3 50%	
	2.64	469	0.184	H-2 → L+4 54%	
				H-1 → L+3 26%	
	2.83	437	0.161	H-2 → L+5 84%	
II	3.91	317	0.341	H-3 → L+5 44%	**LLCT**
				H-5 → L+6 27%	
	3.92	316	0.106	H-3 → L+6 42%	
				H-5 → L+5 41%	
	4.16	298	0.122	H-1 → L+10 31%	
				H-9 → L+2 19%	
	4.19	296	0.244	H-9 → L+2 35%	
	4.27	290	0.248	H-7 → L+5 30%	
				H-8 → L+6 15%	
	4.31	288	0.101	H-5 → L+5 19%	
				H-7 → L+2 19%	
	4.32	287	0.173	H-8 → L+2 21%	
				H-7 → L+4 9%	
				H-8 → L+3 9%	
	4.33	286	0.120	H-1 → L+11 33%	
				H-7 → L+2 22%	
	4.37	284	0.158	H-1 → L+12 19%	
				H-8 → L+2 14%	
				H-1 → L+11 10%	
	4.38	283	0.113	H-1 → L+12 19%	
				H-8 → L+2 14%	
				H-1 → L+11 10%	
	4.44	279	0.194	H-18 → L+2 25%	
				H-8 → L+2 14%	
				H-1 → L+12 6%	
	4.50	275	0.226	H-7 → L+4 32%	
				H-8 → L+3 12%	
				H-9 → L+3 8%	

**ΔE, *f***	***hole***	***particle***
**S1**<br>2.26<br>*0.004*	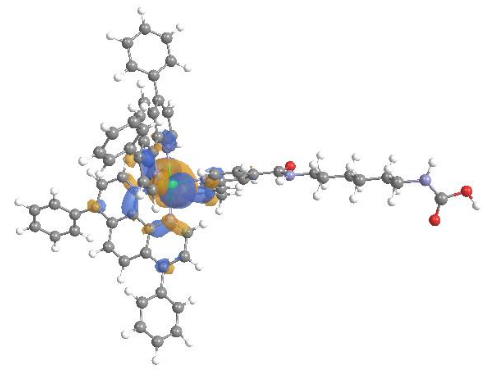	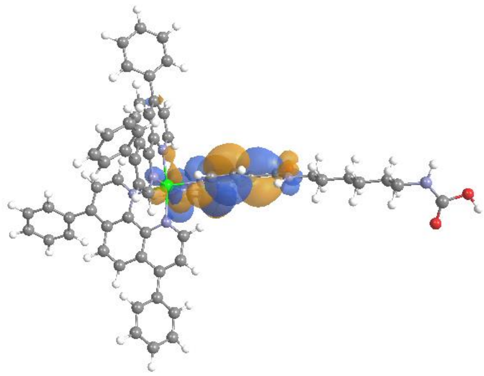
**S2**<br>2.37<br>*0.009*	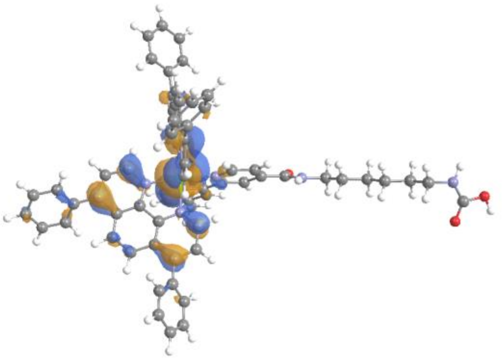	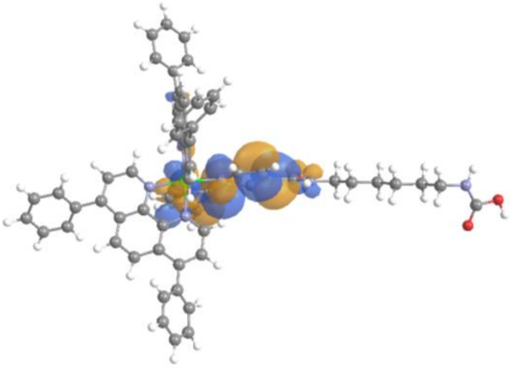
**S3**<br>2.41<br>0.007	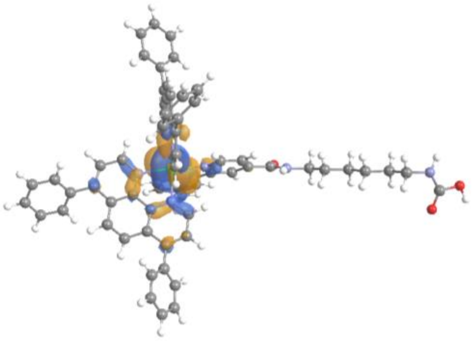	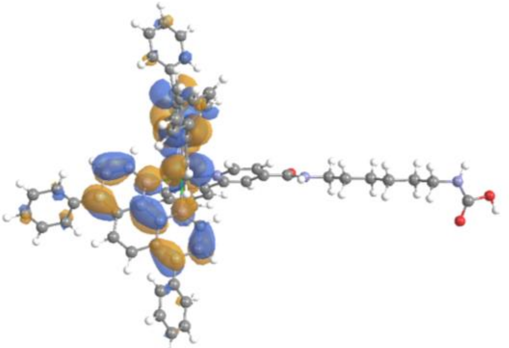
**S4**<br>2.42<br>0.001	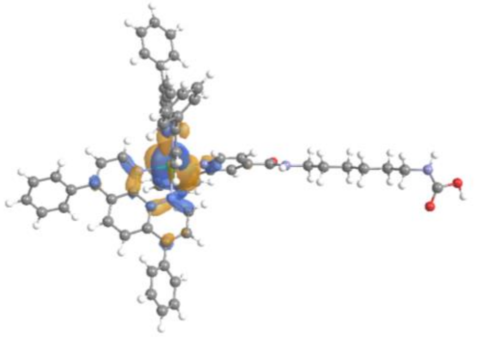	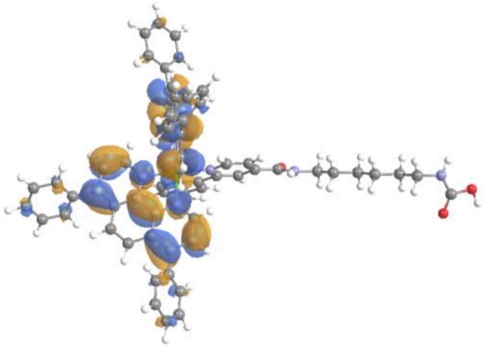

**S5**<br>2.51<br>*0.107*	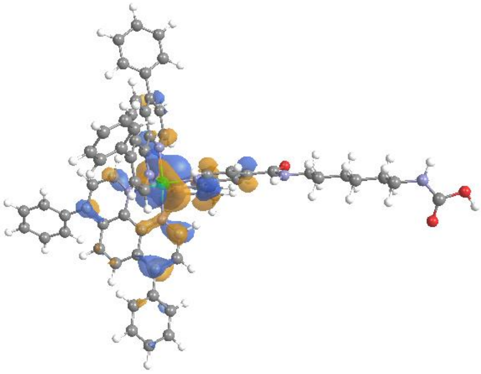	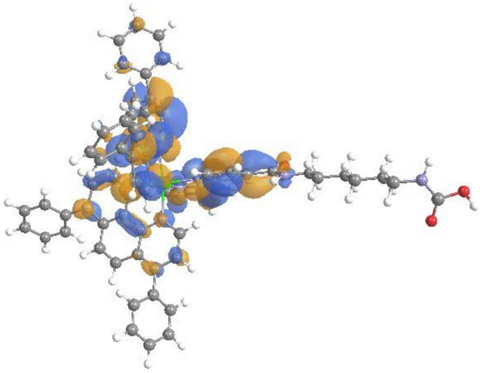
**S6**<br>2.52<br>0.097	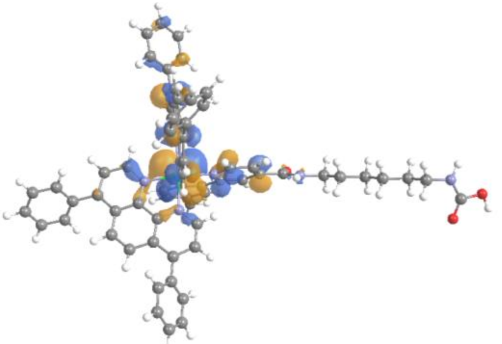	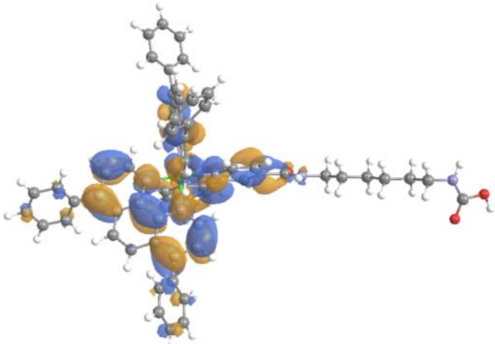
**S7**<br>2.62<br>0.099	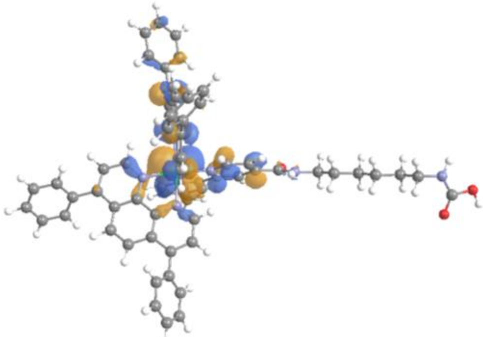	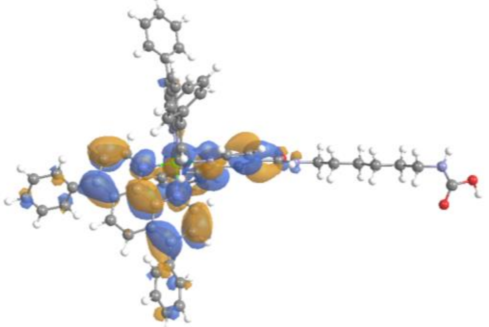
**S8**<br>2.64<br>*0.192*	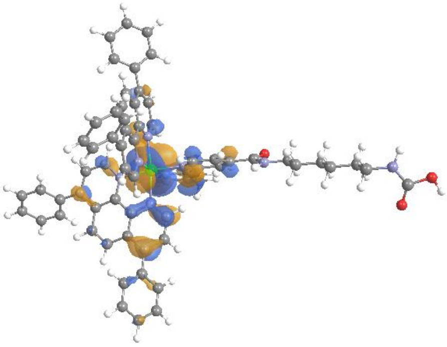	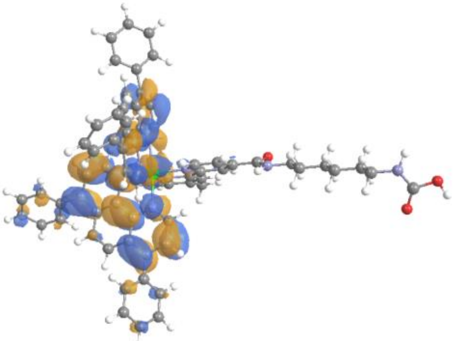
2.83<br>*0.150*	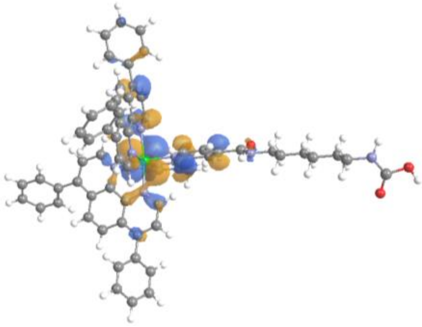	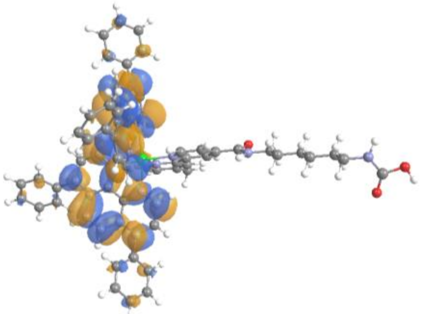

3.91<br>*0.267*	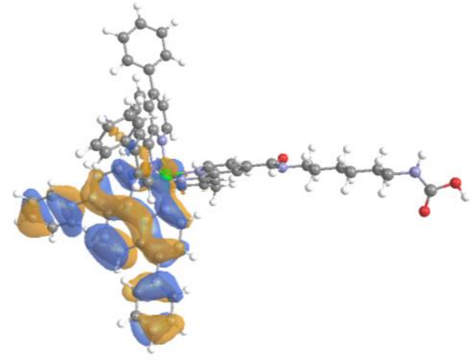	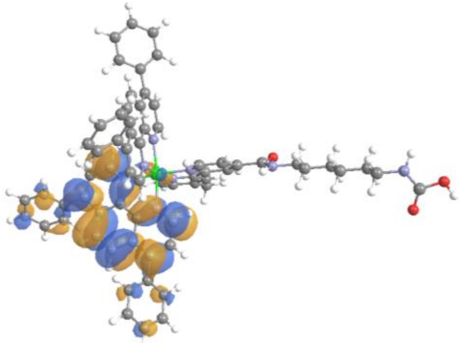
3.92<br>*0.177*	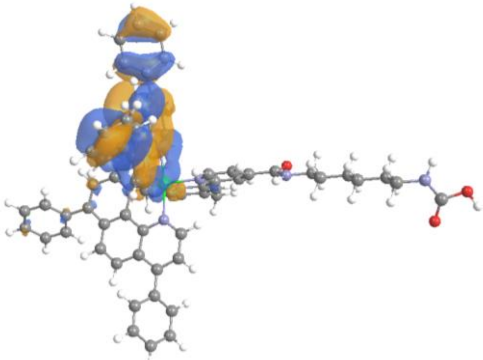	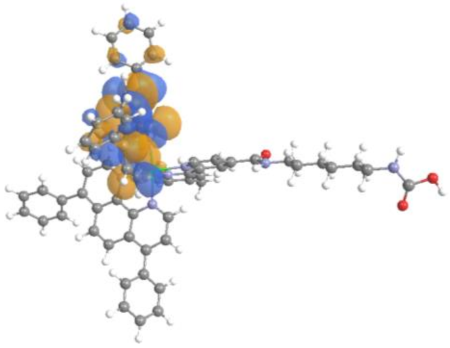
4.17<br>*0.163*	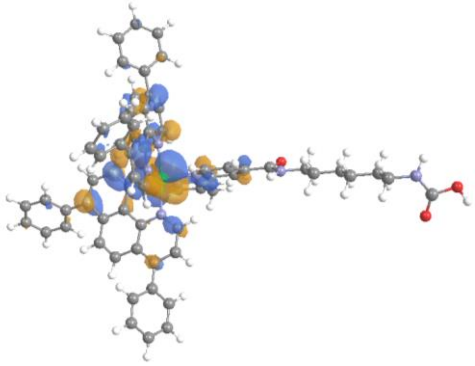	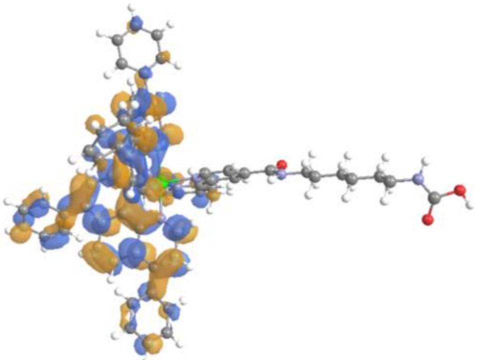
4.19<br>*0.222*	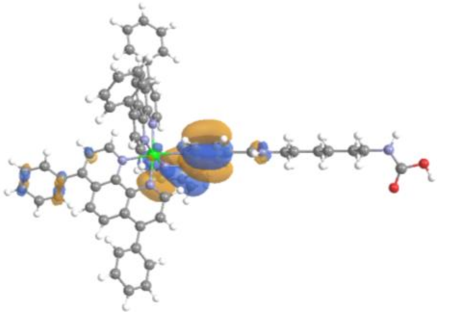	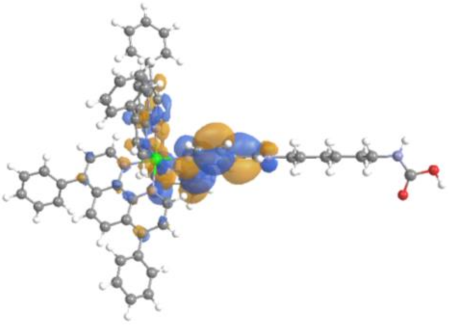
4.27<br>*0.252*	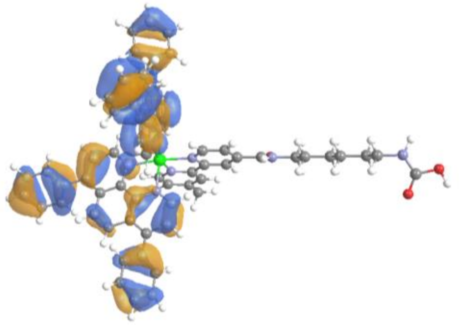	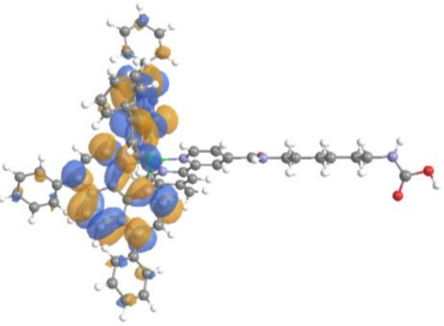

4.32<br>*0.164*	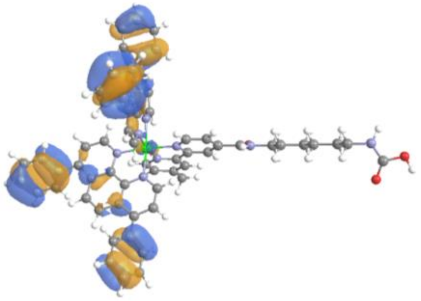	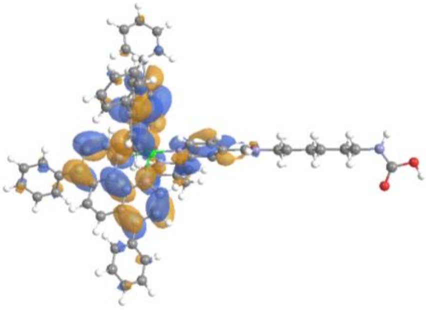
4.33<br>*0.139*	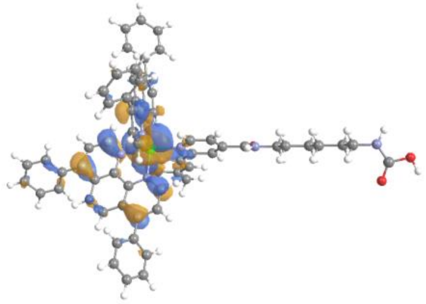	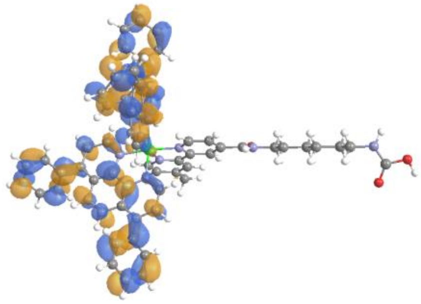
4.37<br>*0.179*	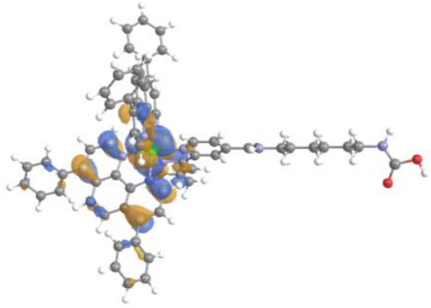	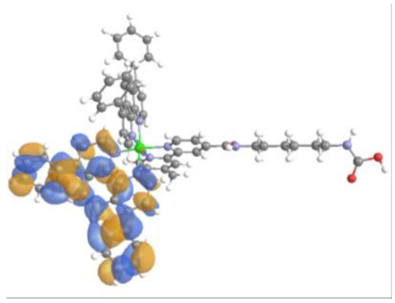
4.39<br>*0.137*	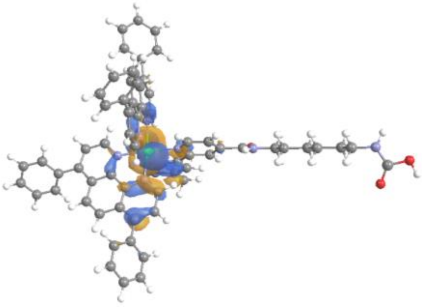	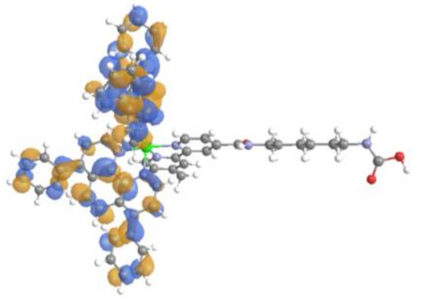
4.43<br>0.146	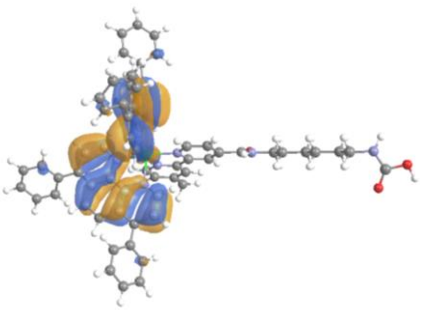	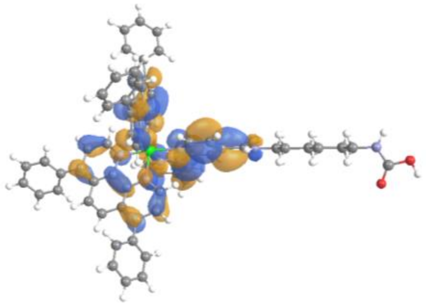

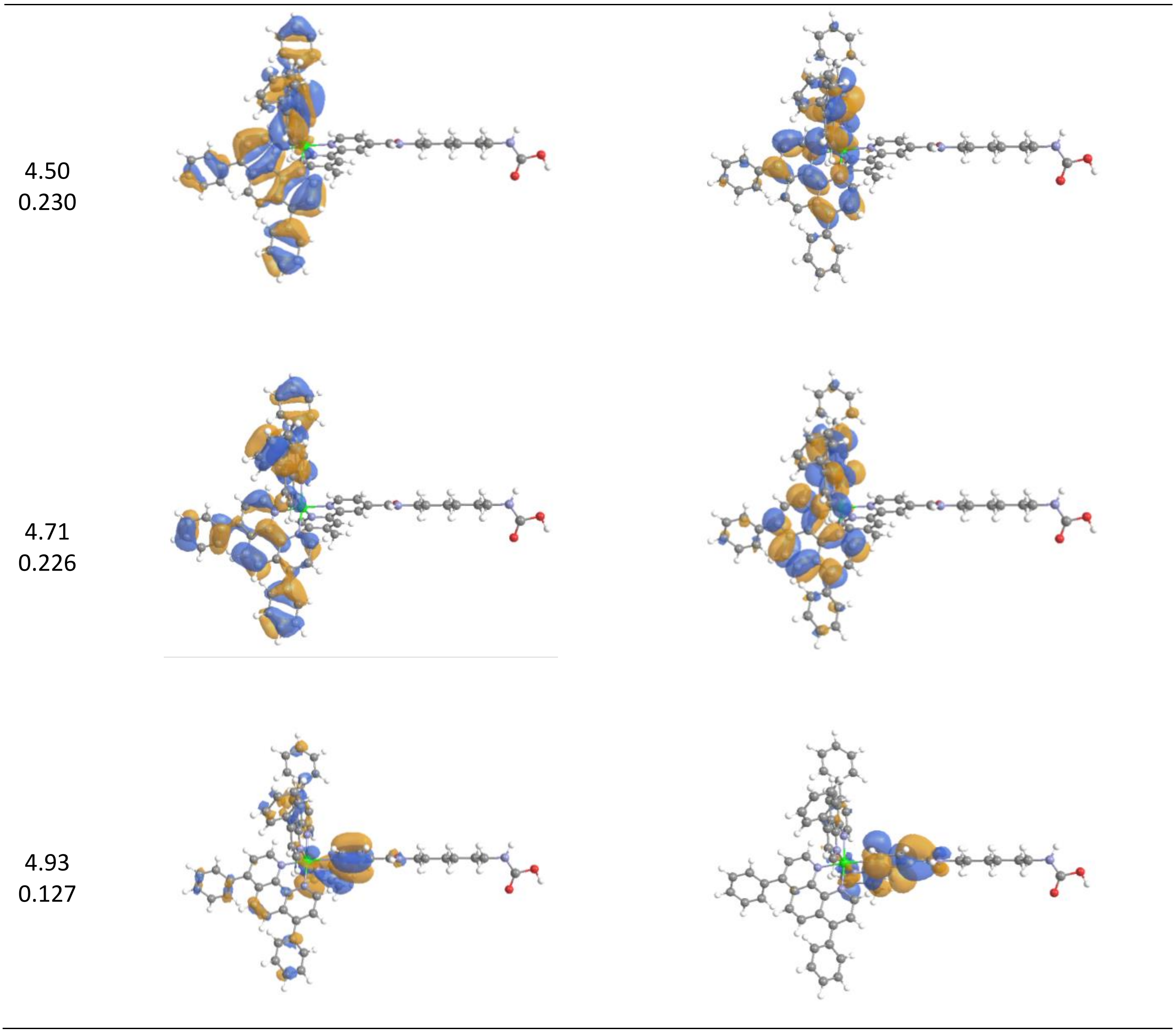

Figure S5: Vertical electronic excitations **ΔE** in eV of **Ru^{II}** complex and NTOs for the excited states with oscillator strength greater than 0.1.

**ΔE, *f***	***hole***	***particle***
**S1**<br>2.27<br>0.004	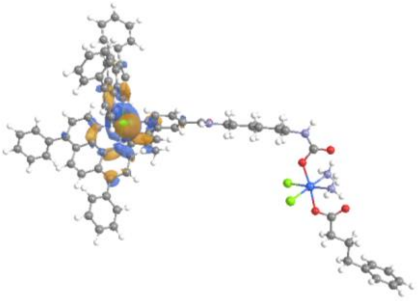	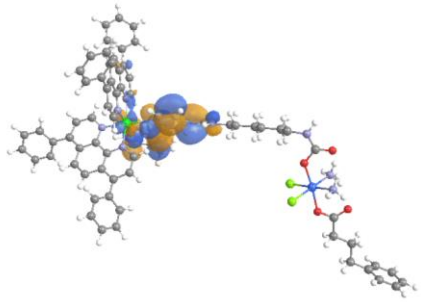
**S2**<br>2.37<br>0.010	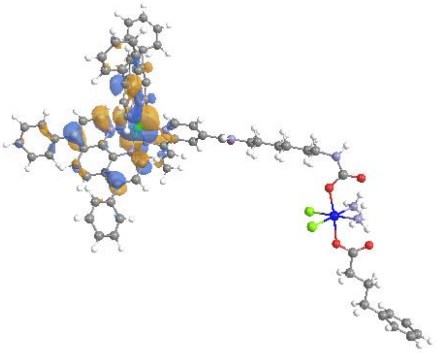	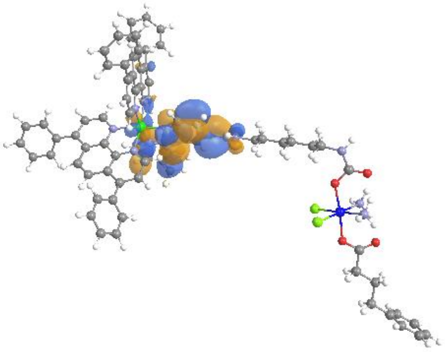
**S3**<br>2.41<br>0.006	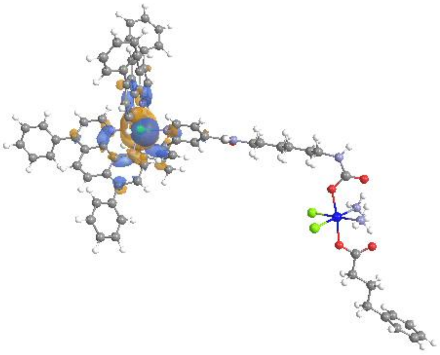	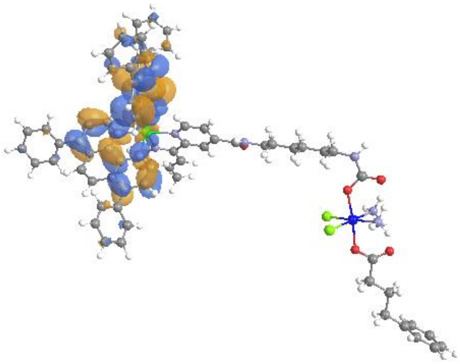
**S4**<br>2.43<br>0.001	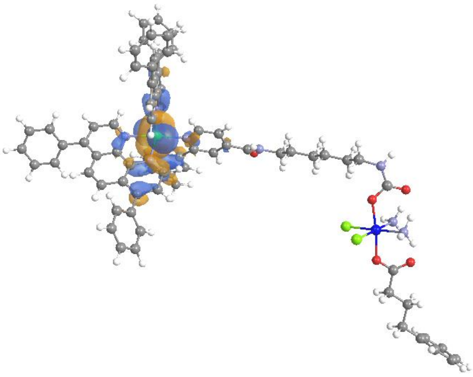	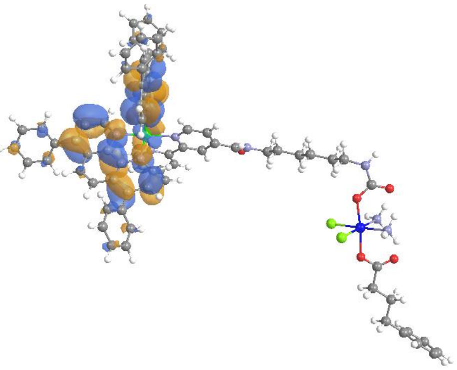

**S5**<br>2.51<br>0.089	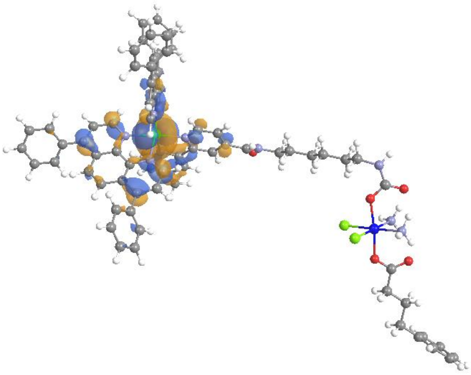	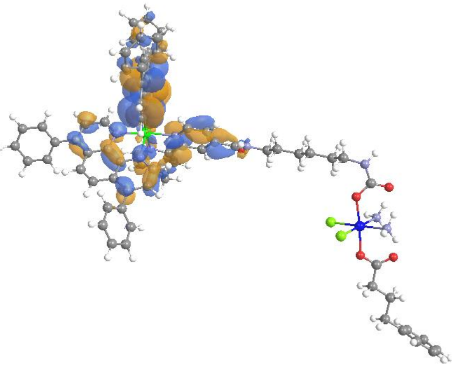
**S6**<br>2.52<br>*0.118*	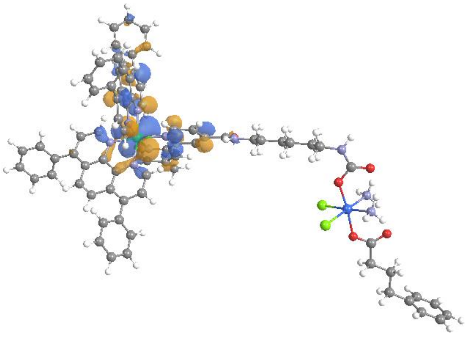	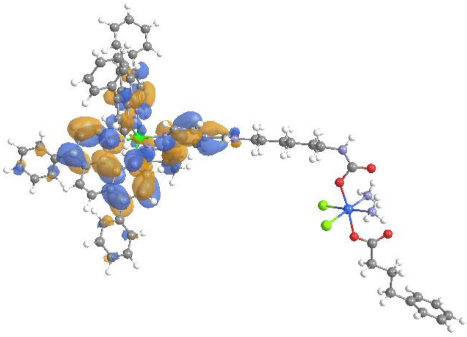
**S7**<br>2.62<br>*0.113*	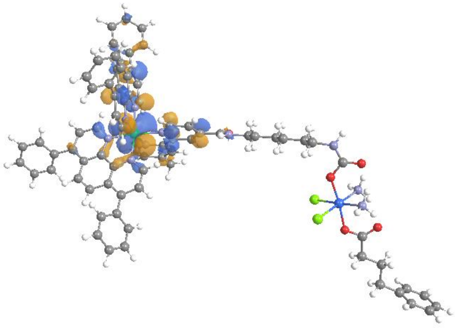	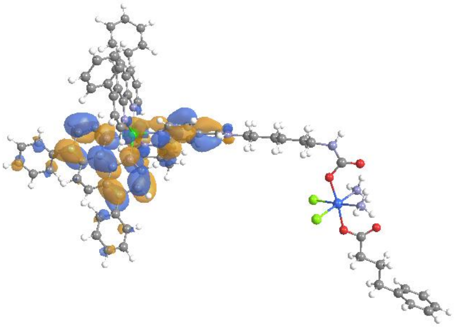
**S8**<br>2.64<br>*0.150*	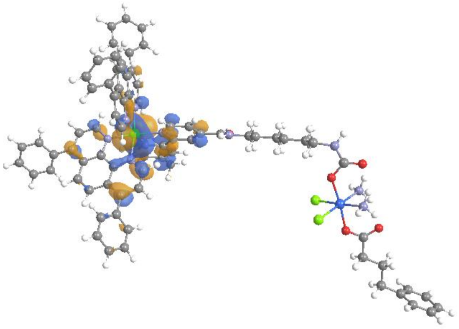	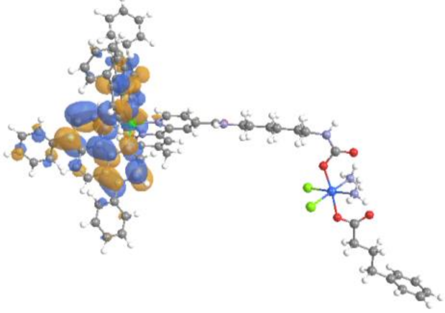
2.83<br>*0.161*	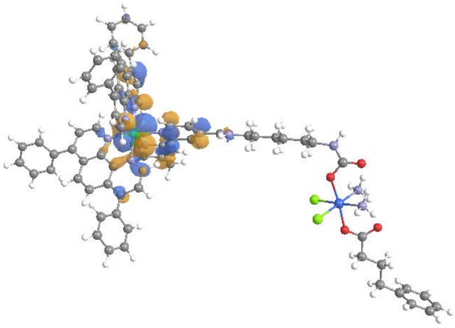	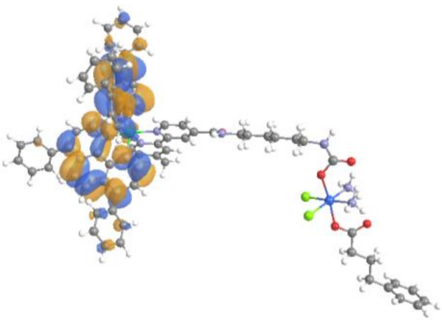

3.91<br>*0.341*	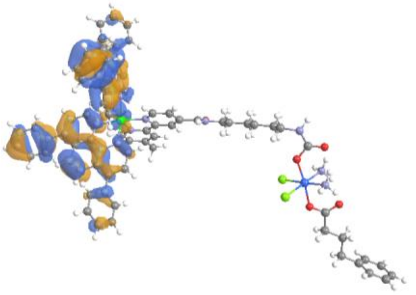	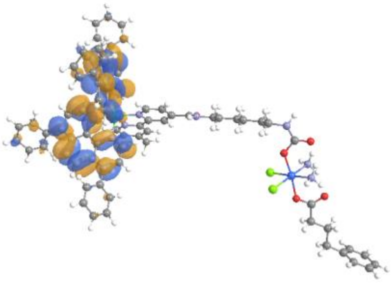
3.92<br>*0.106*	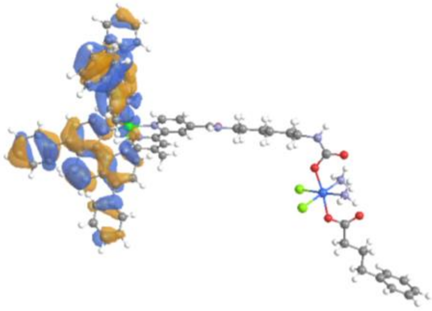	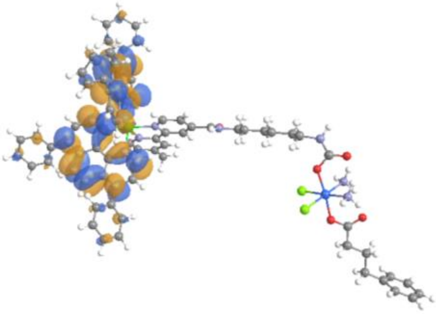
4.16<br>*0.122*	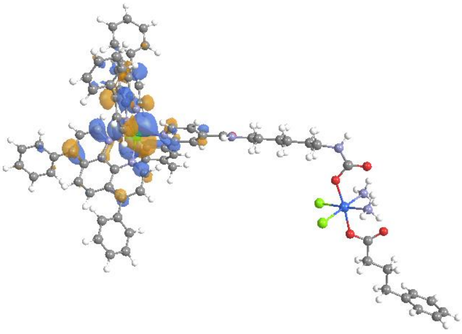	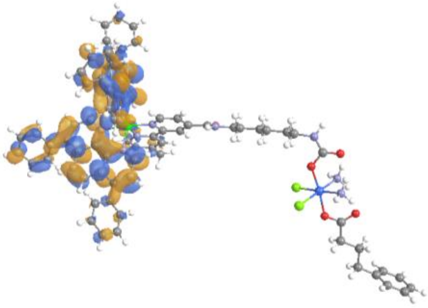
4.19<br>*0.244*	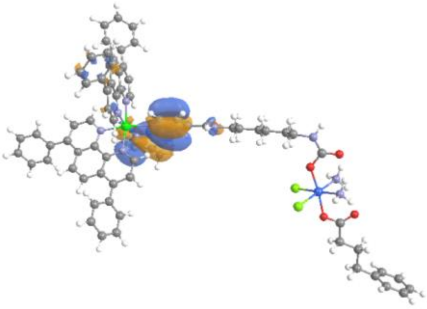	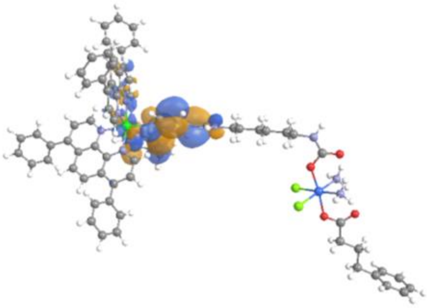
4.27<br>*0.248*	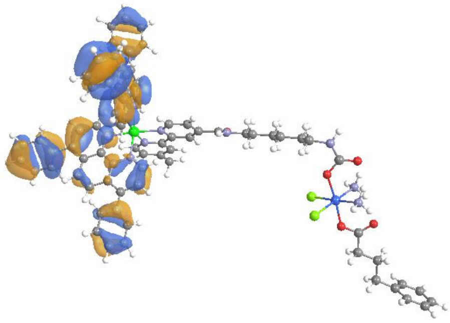	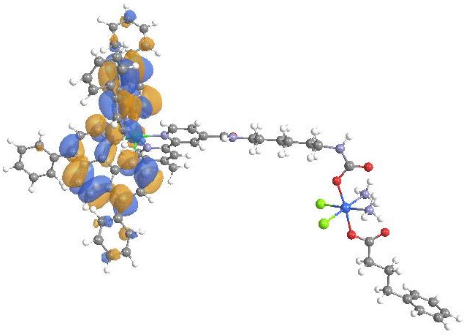

4.31
0.101

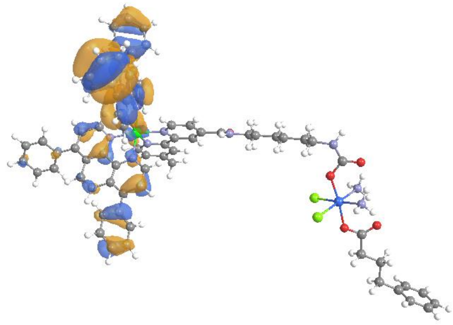

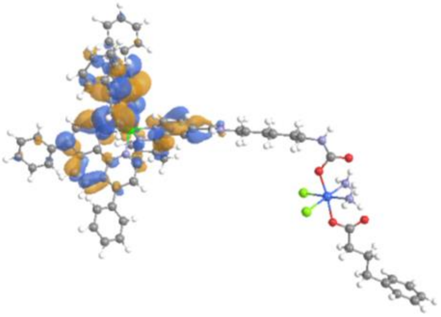

4.32
0.173

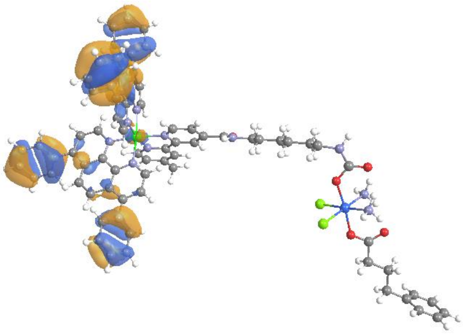

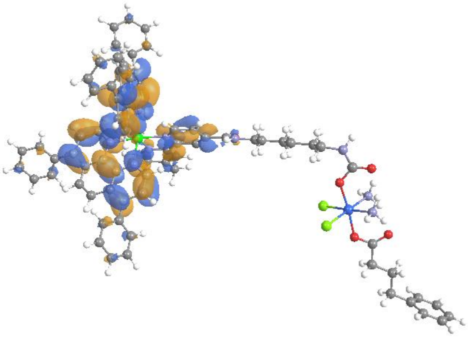

4.33
0.120

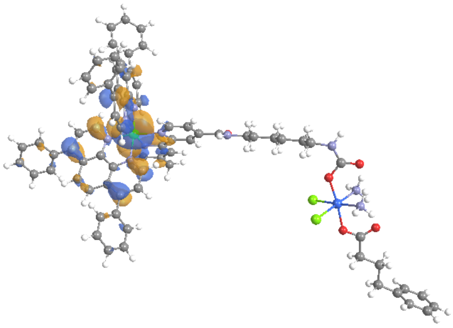

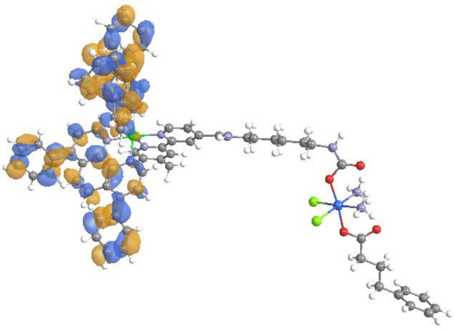

4.37
0.158

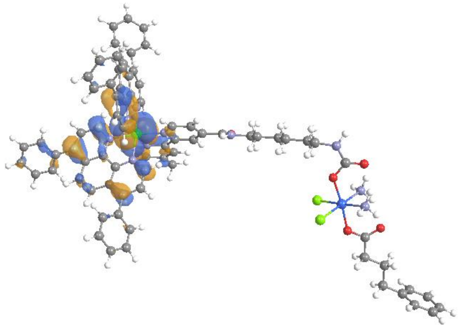

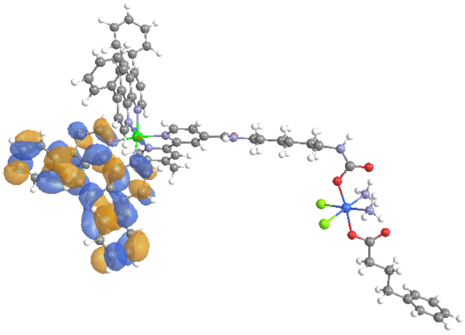

4.38
0.113

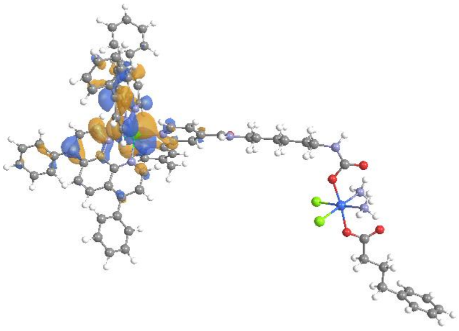

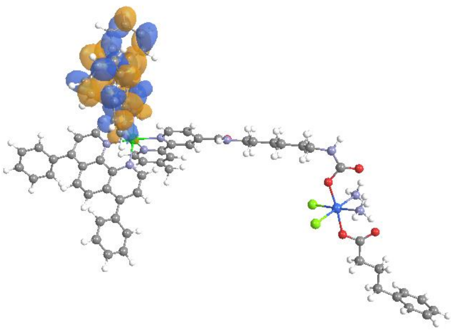

4.44
0.194

4.50
0.226

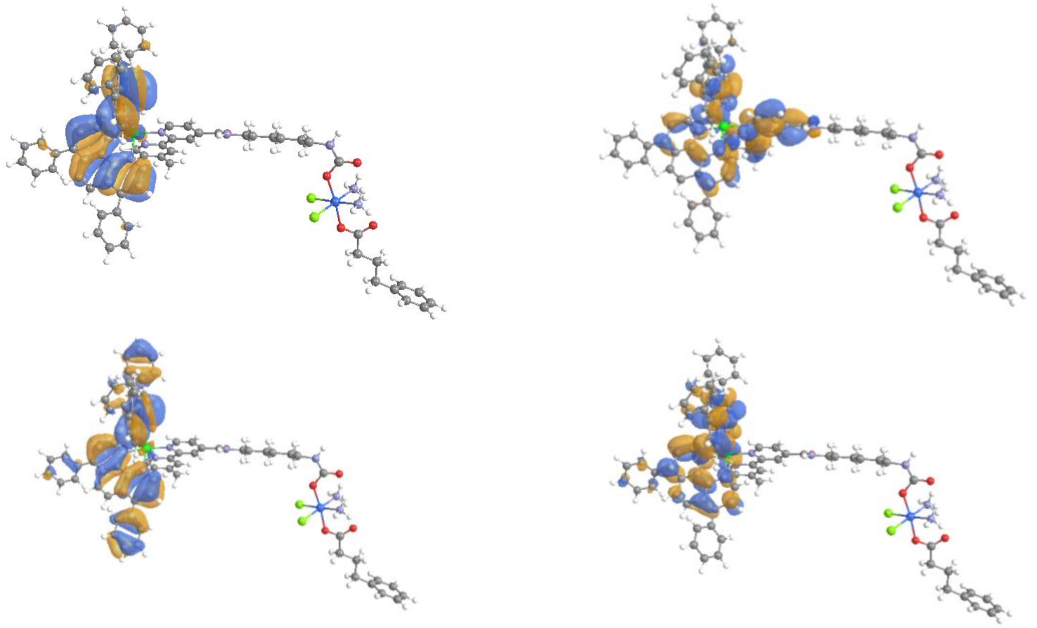

Figure S6: Vertical electronic excitations **ΔE** in eV of **Ru^{II}-Pt^{IV}** assembly and NTOs for the excited states with oscillator strength greater than 0.1.

Table S3: Triplet states excitation energies (eV), MO contribution (%) and theoretical assigned character for **Ru**^{II} complex.

State	ΔE	MO contribution	Theoretical Assignment
T1	2.10	H → L 28%	$ML_1CT/L_{2-3}L_1CT$
		H-2 → L 25%	
		H-2 → L+1 22%	
T2	2.15	H → L 23%	$ML_1CT/L_{2-3}L_1CT$
		H → L+1 21%	
		H-2 → L+1 18%	
T3	2.20	H → L+2 41%	$ML_{2-3}CT$
		H-1 → L+3 14%	
		H → L+1 13%	
T4	2.21	H → L 24%	$ML_{2-3}CT$
		H → L+1 23%	
T5	2.24	H-1 → L+2 41%	$ML_{1-3}CT$
		H-2 → L 28%	
T6	2.28	H-1 → L+1 63%	$ML_{1-3}CT$
		H-2 → L+2 20%	
T7	2.29	H-1 → L 77%	ML_1CT
T8	2.43	H → L+2 23%	$ML_{2-3}CT$
		H-1 → L+3 17%	
		H-2 → L+1 13%	
T9	2.47	H-2 → L+2 40%	$ML_{2-3}CT/L_1L_{2-3}CT$
		H → L+1 11%	
T10	2.56	H-1 → L+3 20%	$ML_{2-3}CT$
		H-2 → L+1 17%	
		H-1 → L+4 12%	
T11	2.57	H-2 → L+3 22%	$ML_{2-3}CT$
		H-1 → L+4 18%	
		H-1 → L+2 13%	
T12	2.63	H → L+3 74%	$ML_{2-3}CT$

Table S4: Triplet states excitation energies eV), MO contribution (%) and theoretical assigned character for **Ru^{II}-Pt^{IV}** assembly.

State	ΔE	MO contribution	Theoretical Assignment
T1	2.11	H → L+2 28%	$ML_1CT/L_{2-3}L_1CT$
		H-2 → L+3 23%	
		H-2 → L+2 23%	
T2	2.16	H → L+3 26%	$ML_1CT/L_{2-3}L_1CT$
		H → L+2 26%	
		H-2 → L+3 18%	
T3	2.20	H → L+4 37%	$ML_{2-3}CT$
		H-1 → L+4 16%	
		H → L+3 10%	
T4	2.21	H → L+2 23%	$ML_{2-3}CT$
		H → L+3 21%	
T5	2.24	H-1 → L+4 41%	$ML_{2-3}CT$
		H-2 → L+2 31%	
T6	2.28	H-1 → L+3 56%	$ML_{2-3}CT$
		H-2 → L+4 16%	
		H-1 → L+2 11%	
T7	2.30	H-1 → L+2 72%	$ML_1CT/L_{2-3}L_1CT$
		H-1 → L+3 18%	
T8	2.42	H → L+4 39%	$ML_{2-3}CT$
		H-2 → L+2 14%	
		H-2 → L+3 14%	
T9	2.47	H-2 → L+4 38%	$ML_{2-3}CT/L_1L_{2-3}CT$
		H → L+3 16%	
T10	2.56	H-1 → L+5 27%	$ML_{2-3}CT$
		H-2 → L+3 17%	
		H-2 → L+4 13%	
		H-1 → L+6 13%	
T11	2.56	H-1→ L+6 20%	$ML_{2-3}CT/L_1L_{2-3}CT$
		H-1 → L+5 18%	
		H-2 → L+4 16%	
T12	2.63	H → L+5 76%	$ML_{2-3}CT$

Table S5. SOC values (cm^{-1}) for the $S_n \rightarrow T_m$ (with n = 1-8) radiationless transitions and singlet–triplet energy gaps (eV) computed for **Ru^II^** complex.

	$S_1 \rightarrow T_m$		$S_2 \rightarrow T_m$		$S_3 \rightarrow T_m$		$S_4 \rightarrow T_m$	
m	*SOC*	*ΔE*	*SOC*	*ΔE*	*SOC*	*ΔE*	*SOC*	*ΔE*
1	228.7	0.16	325.4	0.27	41.4	0.31	68.3	0.32
2	253.2	0.11	249.2	0.22	107.4	0.26	161.8	0.27
3	360.5	0.06	108.2	0.17	28.7	0.21	10.1	0.22
4	63.9	0.05	46.6	0.16	56.7	0.20	71.5	0.21
5	71.5	0.02	115.2	0.12	16.1	0.17	79.6	0.18
6			132.5	0.09	155.1	0.13	215.4	0.14
7			44.8	0.07	291.4	0.12	161.9	0.13
	$S_5 \rightarrow T_m$		$S_6 \rightarrow T_m$		$S_7 \rightarrow T_m$		$S_8 \rightarrow T_m$	
1	47.1	0.41	149.9	0.42	81.4	0.54	177.7	0.52
2	105.8	0.36	56.7	0.37	82.7	0.49	147.8	0.47
3	72.1	0.31	180.1	0.32	47.3	0.44	169.2	0.43
4	147.4	0.30	35.7	0.31	251.6	0.43	100.5	0.41
5	210.1	0.27	263.4	0.28	152.4	0.40	89.5	0.38
6	180.5	0.23	115.9	0.24	141.7	0.36	226.0	0.34
7	108.1	0.21	125.3	0.23	45.4	0.35	198.5	0.33
8	176.9	0.08	58.5	0.09	231.2	0.21	147.4	0.19
9	137.6	0.04	159.3	0.05	121.3	0.17	138.2	0.15
					165.1	0.09	30.5	0.07
					41.1	0.08	88.0	0.06
					30.0	0.02	18.1	0.00

Table S6. SOC values (cm^{-1}) for the $S_n \rightarrow T_m$ (with n = 1-8) radiationless transitions and singlet–triplet energy gaps (eV) computed for the pro-drug **Ru^{II}-Pt^{IV}**.

	$S_1 \rightarrow T_m$		$S_2 \rightarrow T_m$		$S_3 \rightarrow T_m$		$S_4 \rightarrow T_m$	
m	*SOC*	*ΔE*	*SOC*	*ΔE*	*SOC*	*ΔE*	*SOC*	*ΔE*
1	213.2	0.17	162.8	0.26	145.8	0.30	247.4	0.32
2	17.8	0.12	119.0	0.21	110.9	0.25	82.2	0.27
3	85.0	0.07	74.0	0.17	187.6	0.21	164.9	0.22
4	363.3	0.06	33.6	0.16	27.2	0.20	92.7	0.21
5	43.0	0.03	101.0	0.13	95.6	0.17	225.5	0.18
6			72.3	0.09	147.4	0.13	217.8	0.15
7			278.4	0.07	187.8	0.11	30.5	0.13
8							75.6	0.00
	$S_5 \rightarrow T_m$		$S_6 \rightarrow T_m$		$S_7 \rightarrow T_m$		$S_8 \rightarrow T_m$	
1	109.5	0.40	55.3	0.41	103.2	0.52	131.3	0.53
2	137.0	0.35	77.4	0.36	248.3	0.46	231.8	0.48
3	168.1	0.30	125.2	0.32	151.3	0.42	175.5	0.44
4	29.5	0.29	86.5	0.31	143.8	0.41	200.4	0.43
5	216.1	0.26	250.0	0.28	87.9	0.38	105.8	0.40
6	125.8	0.23	74.7	0.24	160.2	0.34	118.8	0.36
7	85.1	0.21	231.3	0.22	72.3	0.32	108.9	0.34
8	180.3	0.08	59.2	0.10	185.3	0.20	188.2	0.22
9	140.2	0.03	184.9	0.05	135.9	0.15	93.4	0.17
10					137.0	0.07	69.6	0.08
11					51.6	0.06	80.8	0.08
12							26.6	0.01

**ΔE, *f***	***hole***	***particle***
**T1**<br>2.10	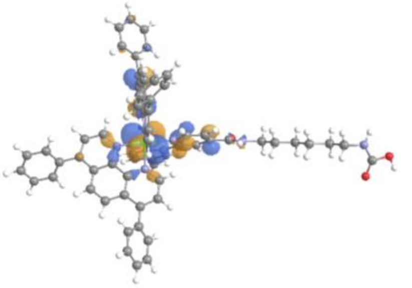	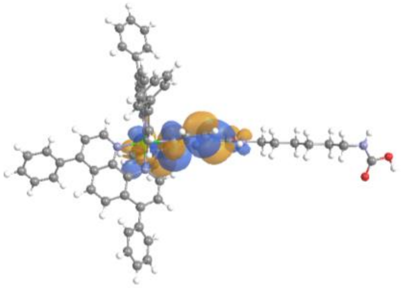
**T2**<br>2.15	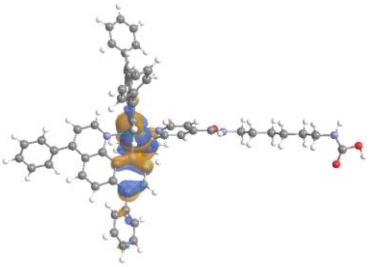	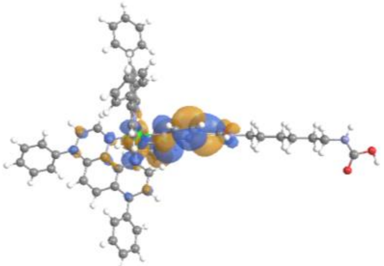
**T3**<br>2.20	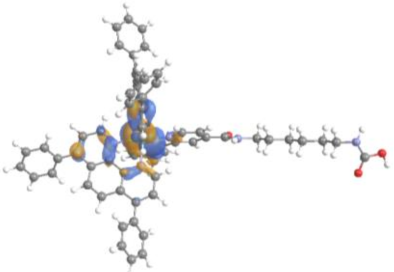	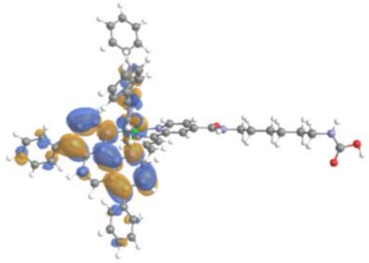
**T4**<br>2.21	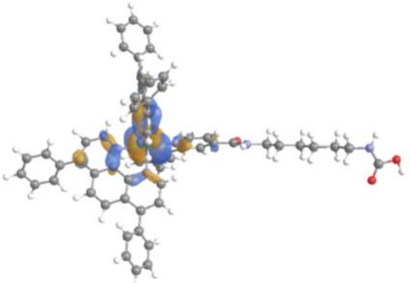	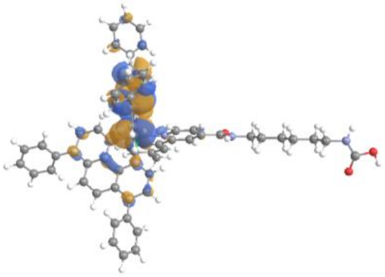
**T5**<br>2.24	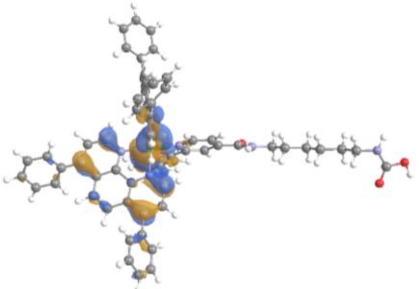	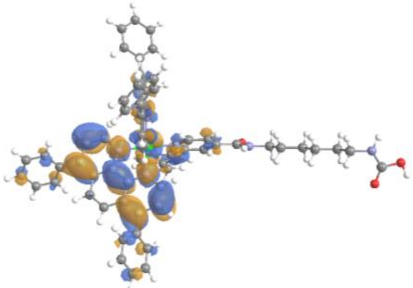
**T6**<br>2.28	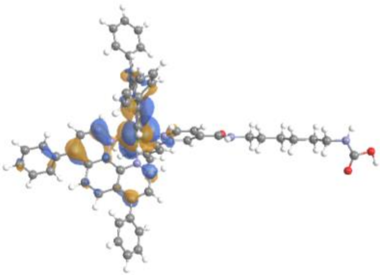	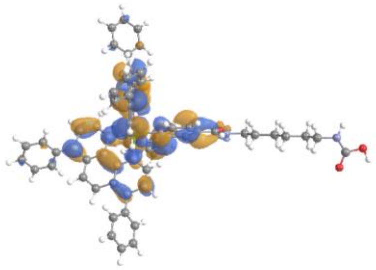

T7
2.29

T8
2.43

T9
2.47

T10
2.56

T11
2.57

T12
2.63

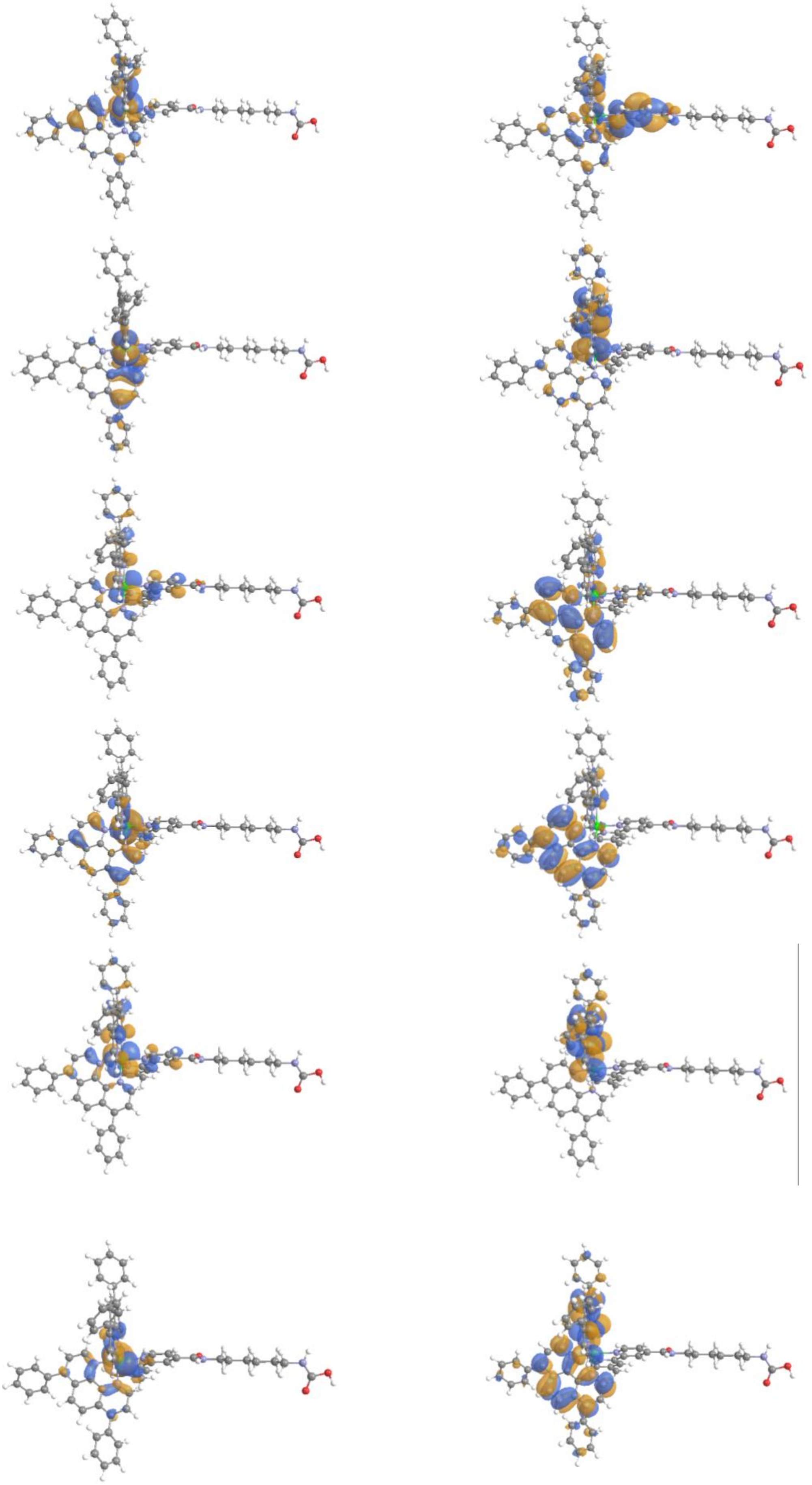

Figure S7: NTOs for the excited triplet states of **Ru^{II}** complex.

**ΔE, *f***	***hole***	***particle***
**T1**<br>2.11	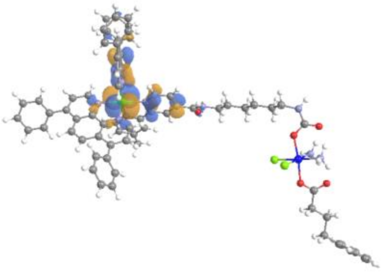	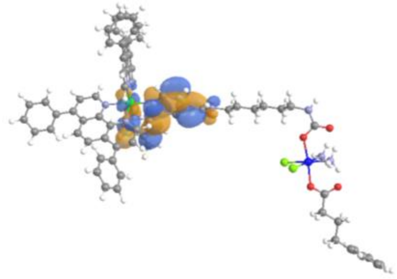
**T2**<br>2.16	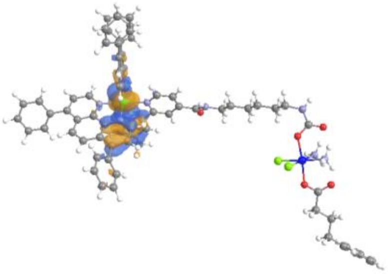	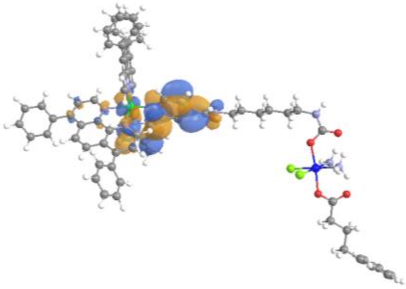
**T3**<br>2.20	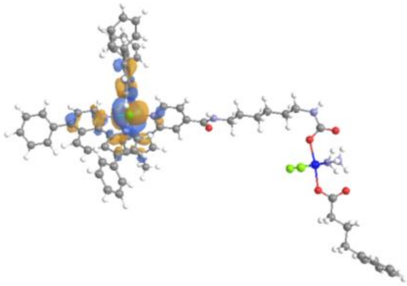	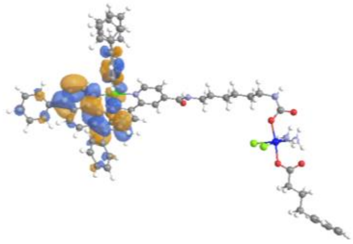
**T4**<br>2.21	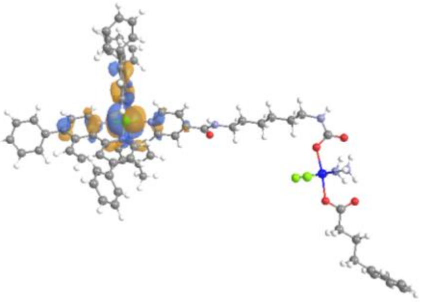	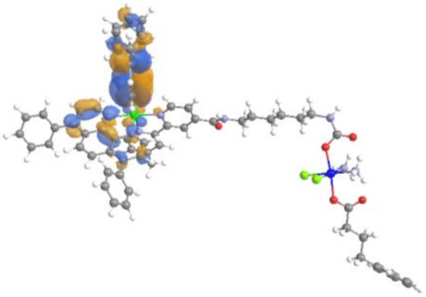
**T5**<br>2.24	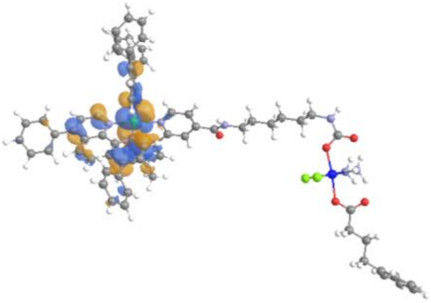	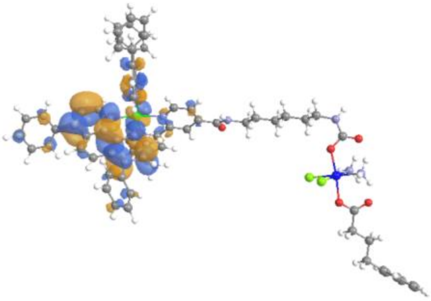
**T6**<br>2.28	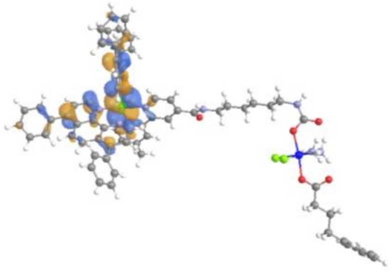	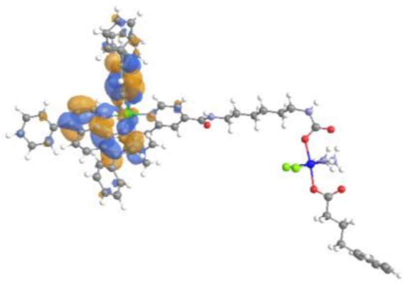

T7
2.30

T8
2.42

T9
2.47

T10
2.56

T11
2.56

T12
2.63

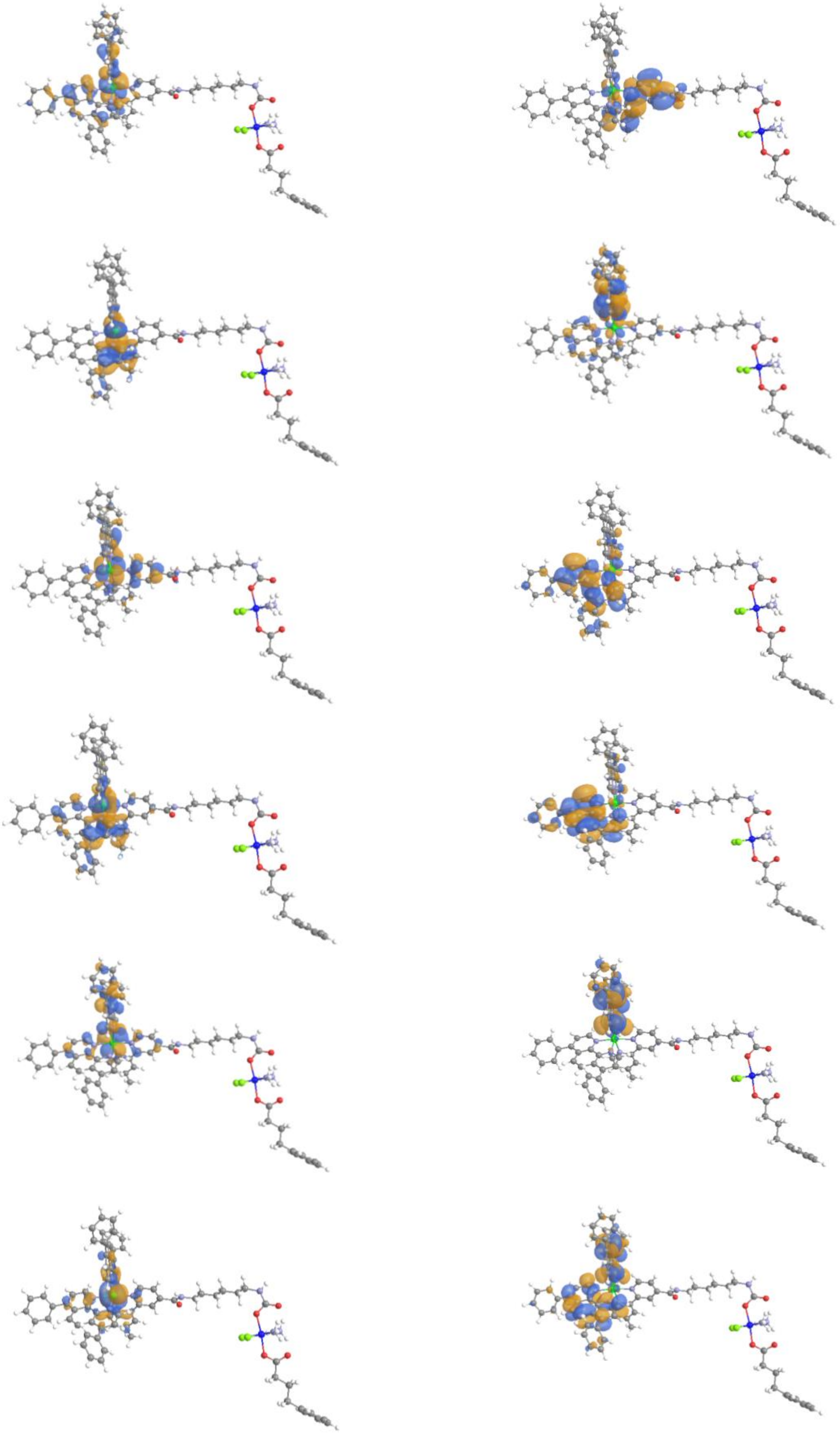

Figure S8: NTOs for the excited triplet states of **Ru^II^-Pt^IV^** complex.
